# Analysis of gene expression dynamics and differential expression in viral infections using generalized linear models and quasi-likelihood methods

**DOI:** 10.3389/fmicb.2024.1342328

**Published:** 2024-04-09

**Authors:** Mostafa Rezapour, Stephen J. Walker, David A. Ornelles, Patrick M. McNutt, Anthony Atala, Metin Nafi Gurcan

**Affiliations:** ^1^Center for Artificial Intelligence Research, Wake Forest University School of Medicine, Winston-Salem, NC, United States; ^2^Wake Forest Institute for Regenerative Medicine, Wake Forest University School of Medicine, Winston-Salem, NC, United States; ^3^Microbiology and Immunology, Wake Forest University School of Medicine, Winston-Salem, NC, United States

**Keywords:** 3D airway organ tissue equivalent (OTEs), RNA-seq data, generalized linear models, quasi-likelihood *F*-test, differentially expressed genes

## Abstract

**Introduction:**

Our study undertakes a detailed exploration of gene expression dynamics within human lung organ tissue equivalents (OTEs) in response to Influenza A virus (IAV), Human metapneumovirus (MPV), and Parainfluenza virus type 3 (PIV3) infections. Through the analysis of RNA-Seq data from 19,671 genes, we aim to identify differentially expressed genes under various infection conditions, elucidating the complexities of virus-host interactions.

**Methods:**

We employ Generalized Linear Models (GLMs) with Quasi-Likelihood (QL) F-tests (GLMQL) and introduce the novel Magnitude-Altitude Score (MAS) and Relaxed Magnitude-Altitude Score (RMAS) algorithms to navigate the intricate landscape of RNA-Seq data. This approach facilitates the precise identification of potential biomarkers, highlighting the host’s reliance on innate immune mechanisms. Our comprehensive methodological framework includes RNA extraction, library preparation, sequencing, and Gene Ontology (GO) enrichment analysis to interpret the biological significance of our findings.

**Results:**

The differential expression analysis unveils significant changes in gene expression triggered by IAV, MPV, and PIV3 infections. The MAS and RMAS algorithms enable focused identification of biomarkers, revealing a consistent activation of interferon-stimulated genes (e.g., IFIT1, IFIT2, IFIT3, OAS1) across all viruses. Our GO analysis provides deep insights into the host’s defense mechanisms and viral strategies exploiting host cellular functions. Notably, changes in cellular structures, such as cilium assembly and mitochondrial ribosome assembly, indicate a strategic shift in cellular priorities. The precision of our methodology is validated by a 92% mean accuracy in classifying respiratory virus infections using multinomial logistic regression, demonstrating the superior efficacy of our approach over traditional methods.

**Discussion:**

This study highlights the intricate interplay between viral infections and host gene expression, underscoring the need for targeted therapeutic interventions. The stability and reliability of the MAS/RMAS ranking method, even under stringent statistical corrections, and the critical importance of adequate sample size for biomarker reliability are significant findings. Our comprehensive analysis not only advances our understanding of the host’s response to viral infections but also sets a new benchmark for the identification of biomarkers, paving the way for the development of effective diagnostic and therapeutic strategies.

## 1 Introduction

Organoids, intricate *in vitro* models formed through tissue engineering, faithfully reproduce the complex structure and functionality of corresponding *in vivo* tissues, finding wide-ranging applications in human tissue development, repair, diagnostics, disease modeling, drug discovery, and personalized medicine. Derived from pluripotent or tissue-resident stem cells, organoids emulate diverse tissues, including tumors, and offer a comprehensive platform for investigating tissue biology (Zhao et al., 2022). While conventional methods involve pluripotent stem cell self-assembly (Lehmann et al., 2019), recent innovations have introduced novel approaches. Wang et al. (2022) explored patient-derived organoid cultures to study colorectal cancer (CRC) mechanisms, revealing how culture methods influence gene expression patterns. Maiti et al. (2022) dissected human cornea organoids and donor corneas, elucidating early developmental states and their relevance for corneal diseases. Kathuria et al. (2020) generated cerebral organoids from individuals with bipolar disorder, uncovering gene expression differences and synaptic impairments. Pei et al. (2021) employed lung organoids to study SARS-CoV-2 infection, revealing virus-cell interactions and testing therapeutic interventions. Integrating adult stem cells and matrices, micro-scale organ-tissue equivalents (Huh et al., 2011) replicate specific tissue characteristics, accelerating functional maturation and enabling interactions unique to each organ (Drost and Clevers, 2017; Oksdath et al., 2018; Valdoz et al., 2021; Blatchley and Anseth, 2023). Commercial sources and tissue-specific media continue to expand organ-tissue equivalents possibilities (Hofer and Lutolf, 2021).

Bhowmick et al. (2018) developed a 3D Human Tissue-Engineered Lung Model (3D-HTLM) to study Influenza A Virus (IAV) pathogenesis and the immune response, addressing a critical gap in influenza research. By culturing primary human small airway epithelial cells (HSAEpCs) on a 3D chitosan-collagen scaffold, they aimed to more closely mimic the human lung’s complex architecture. Their findings demonstrated that 3D-cultured HSAEpCs exhibited a more robust response to IAV infection than their 2D counterparts, including significant differences in cell viability, morphology, and cytokine release, thereby underscoring the importance of 3D models in replicating *in vivo*-like responses.

Similarly, Zhou et al. (2018) utilized 3D human airway organoids to improve the assessment of influenza virus infectivity. Their work highlighted the organoids’ ability to closely mimic the human airway epithelium, enabling the differentiation of ciliated, goblet, club, and basal cells to nearly physiological levels. This advancement proved crucial for evaluating the infectivity of emerging influenza viruses, with the differentiated organoids showing greater replicative capabilities for human-infective viruses compared to traditional models.

Kinder et al. (2020) focused on Respiratory Syncytial Virus (RSV) and Human Metapneumovirus (MPV), employing 3D human airway epithelial (HAE) tissues to reveal notable disparities in infection capabilities and mechanisms of spread between the two viruses. This study not only demonstrated the superior infection efficiency and viral release by RSV compared to MPV but also highlighted the potential of 3D models in uncovering unique viral spread mechanisms, such as MPV’s use of actin-based filamentous extensions for cell-to-cell transmission.

Ribó-Molina et al. (2024) leveraged an organoid-derived bronchial culture model to study MPV, offering insights into the virus’s preferential targeting of ciliated epithelial cells and the resultant cilia damage. This model’s close simulation of human respiratory tract conditions provided a more accurate platform for evaluating viral replication and pathogenesis, emphasizing the superiority of 3D organoids in mirroring *in vivo* conditions.

Rijsbergen et al. (2022) and Shpichka et al. (2022) further validated the efficacy of 3D organoids in studying viral infections, including Human Parainfluenza Virus Type 3 (PIV3). These studies highlighted organoids’ capacity to replicate key aspects of viral infections and their utility in exploring infection mechanisms and antiviral treatments, thereby confirming the advantages of 3D models over traditional 2D cultures in infectious disease research.

In the landscape of infectious disease research, the fidelity with which experimental models replicate human tissue complexity significantly influences our understanding of pathogen-host interactions and the efficacy of potential therapeutic interventions. This study introduces an innovative approach by employing upper airway lung Organ-Tissue Equivalents (OTEs) to investigate differential responses to infections by IAV, MPV, and PIV3. Unlike traditional cell culture systems, OTEs offer a sophisticated mimicry of the structural and functional complexity of the human respiratory epithelium, making them a superior model for this investigation.

The advantage of using OTEs lies in their capacity to replicate key features of the upper airway epithelium, such as the presence of diverse cell types including ciliated cells, goblet cells, and basal cells, within a physiologically relevant architecture. This multicellular composition enables a more accurate simulation of virus-host interactions and immune responses within the respiratory tract, thereby offering deeper insights into viral pathogenesis, host defense mechanisms, and the evaluation of therapeutic strategies. Furthermore, OTEs maintain the tissue-level organization and cellular differentiation crucial for understanding the nuanced behavior of respiratory viruses, which exploit specific cell types and tissue structures for entry, replication, and dissemination (Leach et al., 2023).

Central to our study’s methodology is the rigorous fabrication process of OTEs, utilizing non-diseased, non-smoking human lung donor tissue. Through a meticulous decellularization and solubilization process, detailed in our group’s previously published work (Leach et al., 2023), we produce a solubilized lung extracellular matrix (sECM). This sECM, rich in collagen, elastin, sulfated glycosaminoglycans, and hyaluronan, mirrors the native lung ECM composition, providing the essential physical and biochemical cues for the development and maintenance of lung-specific cell types within the OTEs. Advanced fabrication techniques, including the application of thiolated gelatin, hyaluronic acid, and a polyethylene glycol (PEG)-based crosslinker, ensure the OTEs support robust cell–cell and cell-matrix interactions crucial for lung function and response to infections (Leach et al., 2023).

Our study aims to illuminate the differential responses of OTEs to IAV, MPV, and PIV3 infections, focusing on variations in gene expression through a bioinformatics approach tailored to RNA-Seq data encompassing 19,671 genes. These viruses, while sharing a respiratory tropism, induce distinct clinical presentations and immune responses, necessitating a comparative analysis to unravel the complexities of virus-host dynamics. The selection of 24- and 72-h post-infection time points is strategic, capturing early host responses and the progression to adaptive immune mechanisms, respectively.

In our quest to explore the intricate dynamics of gene expression in OTEs infected with various viruses, we opted for RNA-Seq as our primary data collection method over traditional microarray technology. RNA-Seq offers unparalleled depth and breadth in transcriptome analysis, providing the sensitivity to detect a wide range of expression levels and the specificity to uncover novel transcripts and splice variants. This choice aligns with our study’s objective to capture the complex and nuanced biological responses to viral infections, where the detailed characterization of gene expression changes is pivotal. Furthermore, the digital nature of RNA-Seq data, despite not being inherently continuous, is adeptly managed through advanced statistical methods tailored for such high-dimensional count data.

To address the analytical challenges posed by RNA-Seq’s discrete and overdispersed nature, we employ Generalized Linear Models (GLM) (Nelder and Wedderburn, 1972) in conjunction with Quasi-Likelihood (QL) *F*-tests (Wedderburn, 1974), facilitated by the EdgeR (Robinson et al., 2010) package. Our preference for EdgeR over other available tools, such as DESeq2 (Love et al., 2014), stems from its robust handling of biological variability and its flexibility in modeling complex experimental designs. EdgeR’s empirical Bayes methods (Robinson et al., 2010) are particularly effective in our context, where accurate estimation of dispersion and differential expression across small sample sizes is crucial. This decision ensures the integrity and reliability of our analysis, enabling the nuanced interpretation of gene expression dynamics induced by viral infections. Furthermore, the introduction of Magnitude-Altitude Score (MAS) and Relaxed Magnitude-Altitude Score (RMAS) algorithms allows for focused identification of genes significantly impacted by the infection. Gene Ontology (GO) enrichment analysis (Chen et al., 2013) then interprets the biological relevance of these findings, categorizing genes into groups reflective of the host’s transcriptional adjustments in response to viral infection.

## 2 Materials and methods

Our team has established an OTE model of the airway at an air-liquid interface (ALI) to accurately represent the intricate nature of human airways (Leach et al., 2023). This model captures the dynamics of cell interactions, the presence of extracellular matrix (ECM) proteins, and various biomechanical aspects, offering a comprehensive platform for studying respiratory viruses’ interactions with the human airway epithelium. Building on this model’s capabilities, we proceeded to infect the OTEs with three distinct viruses as part of our study. These included IAV strain A/Puerto Rico/8/1934 (H1N1), which features an EGFP-NS1 gene fusion and was kindly provided by Adolfo Garcia-Sastre of the Mount Sinai School of Medicine; MPV with an EGFP gene inserted before the N gene of strain CAN97-83 (product #M121 from ViraTree); and PIV3, which incorporates the EGFP gene between its first (N) and second (P/C/D/V) genes of strain JS (ViraTree product #P323).

For the virus infection, a medium was prepared from Dulbecco-modified Eagle’s minimal essential medium, enriched with 0.1% inactivated fetal bovine serum, 0.3% purified bovine serum albumin, 20 mM HEPES (pH 7.5), and 0.2 mM Glutamax. This medium was sterilized using a 0.2 μm filter and preserved at 4°C. We determined influenza virus titers using Madin-Darby Canine Kidney (MDCK) cells (from ATCC, #CCL-34), while titers for other viruses were ascertained using the LLC-MK2 rhesus monkey kidney cell line (from ATCC, #CCL-185). It was estimated that each lung OTE harbored around 1.6 × 10^5^ epithelial cells, and the nominal multiplicity of infection was based on the assumption that these cells were predominantly susceptible to the viruses under investigation.

For the infection process, OTEs were transferred to a 24-well culture plate containing modified PneumaCult ALI Medium (from Stemcell Technologies) and allowed to stabilize at 37°C in a CO2 incubator. Prior to infection, each OTE surface was cleansed with warm Hank’s balanced saline solution. The virus, diluted in iDMEM, was then applied to the OTE’s apical surface. The plate was incubated with gentle rocking for an hour at 37°C in CO2 conditions, followed by standard growth conditions for specific durations, enabling a detailed exploration of viral infection dynamics within this advanced model system.

To facilitate the subsequent analysis of these virus-host interactions, RNA extraction from the OTEs was meticulously performed using the Direct-zol RNA Miniprep Plus Kit. This procedure began with the preparation of samples, followed by cell lysis, RNA purification, and DNase I treatment to eliminate potential DNA contaminants. The purified RNA was then stored at −80°C, ensuring its preservation for in-depth sequencing and gene expression studies. This extraction process is essential for accurately dissecting the complex interplay between the OTE model and the introduced viral pathogens.

To delve into the molecular underpinnings of these interactions, RNA sequencing was paramount. cDNA libraries were crafted from 50 ng of RNA extracts using a NEXTFLEX® Combo-Seq™ mRNA/miRNA Kit and processed on a Sciclone® G3 NGSx Workstation. The libraries underwent quantification with a KAPA Library Quantification Kit and average fragment analysis via a 4200 TapeStation System. Following normalization, sequencing was conducted on an Illumina® NovaSeq 6000 System to generate 76-bp single-end reads, laying the groundwork for in-depth sequence analysis.

Utilizing Partek® Flow® software, raw sequence data were subjected to meticulous analysis. Initial processing involved Cutadapt (Martin, 2011) for trimming adaptors and low-quality bases, followed by alignment and quantification against the hg38 GENCODE reference database employing STAR (Dobin et al., 2013) and an expectation/maximization (E/M) algorithm akin to Xing et al. (2006). This process culminated in the normalization of transcript-level counts to gene-level data using the median-of-ratios method from DESeq2 (Love et al., 2014), with results log2-transformed for clarity. Principal component analysis (PCA) was subsequently applied to elucidate global expression trends across various experimental conditions and temporal stages.

In this study, we meticulously implemented various control conditions, including UV-treated and naïve samples, to bolster the precision of our experimental outcomes and effectively distinguish the effects of viral infection from other experimental variables. The incorporation of these controls was pivotal for affirming the integrity and reliability of our findings. It’s crucial to note, however, that while fundamental to our study’s design, these samples were not included in the differential expression analysis.

UV-treated samples, subjected to ultraviolet (UV) light to inactivate the viruses, provided a critical control for understanding the impact of viral components without their active replication. This approach helped isolate the effects of viral entry and subsequent host cell responses from those induced by viral replication, enabling us to specifically identify how viral infection mechanisms influence gene expression changes, separate from the effects of active viral proliferation within host cells.

Naïve samples, or untreated OTEs, served as internal controls to establish a gene expression baseline across our experiments. These samples were essential for ensuring that any observed gene expression changes were attributable to experimental manipulation, particularly viral infection, rather than to inherent variability or background expression levels.

Despite the indispensable role of UV-treated and naïve samples in establishing baseline and control conditions, our differential expression analysis focused exclusively on the direct effects of active viral infection on gene expression within the OTE models. Consequently, mock samples, which underwent all experimental procedures without virus introduction, were employed as the baseline for pairwise multiple hypothesis testing between actively infected samples. This approach ensured the gene expression changes we identified and analyzed were a direct consequence of active viral infection, offering a precise and focused insight into virus-host interactions.

The strategic exclusion of UV-treated and naïve samples from expression analysis underscored our commitment to methodological rigor, allowing us to maintain a sharp focus on the specific impacts of viral infection. This decision was instrumental in enhancing the clarity, precision, and relevance of our findings, thereby justifying and reinforcing the validity of our experimental approach.

Our primary aim was to investigate differential gene expression in RNA-Seq data from OTE models infected with IAV, MPV, and PIV3 at 24- and 72-h post-infection, covering a comprehensive spectrum of 19,671 genes. After meticulous preparation and infection of the OTE samples, followed by RNA extraction and quality assessment, we employed the Trimmed Mean of M-values (TMM) normalization method to adjust for library size variations and composition effects, a common challenge in RNA-Seq data analysis (Robinson and Oshlack, 2010).

For analytical clarity and precision, we organized infection conditions into categories based on virus type (IAV, MPV, or PIV3), treatment nature (UV-treated or Non-UV/active), and post-infection duration (24 or 72 h), labeled as Condition-Treatment-Time. Actively infected samples were specifically denoted as Virus-active-Time. Crucially, mock-infected samples (Mock-24 and Mock-72) served as the baseline for our pairwise hypothesis testing, allowing us to attribute observed differential gene expression directly to viral infection.

This replication strategy, involving six replicates at both the 24-h and 72-h time points for each condition, significantly bolstered the statistical strength of our results. The detailed organization of these conditions, alongside the pivotal role of mock samples in our analysis, is thoroughly outlined in Table 1. This structured methodology, combined with the strategic use of control samples, enabled an in-depth and accurate exploration of the effects of viral infections under varied conditions, greatly enriching the depth and accuracy of our research outcomes.

**Table 1:** Sixteen infection conditions categorized based on (1) Virus, (2) Treatment, and (3) post-infection time.

**Primary condition**	**Virus**	**Treatment**	**Post-infection time**	**Primary condition**	**Virus**	**Treatment**	**Post-infection Time**
IAV-None-24	IAV	Active	24-h	IAV-None-72	IAV	Active	72-h
MPV-None-24	MPV	Active	24-h	MPV-None-72	MPV	Active	72-h
PIV3-None-24	PIV3	Active	24-h	PIV3-None-72	PIV3	Active	72-h
Mock-24	Mock	–	24-h	Mock-72	Mock	–	72-h

**In-house controls**	**Virus**	**Treatment**	**Post-infection Time**	**In-house controls**	**Virus**	**Treatment**	**Post-infection Time**
IAV-UV-24	IAV	UV	24-h	IAV-UV-72	IAV	UV	72-h
MPV-UV-24	MPV	UV	24-h	MPV-UV-72	MPV	UV	72-h
PIV3-UV-24	PIV3	UV	24-h	PIV3-UV-72	PIV3	UV	72-h
Untreated/Naïve −24	–	–	24-h	Untreated/ Naïve −72	–	–	72-h

### 2.1 Statistical methodology and gene ontology analysis

To ensure clarity and accessibility, we first define our thresholds for statistical significance as follows:

- Significance (*p* < 0.05): Initially, findings with *p*-values less than 0.05 are considered significant. This stage identifies potential differentially expressed genes (DEGs) before adjusting for the impact of conducting multiple tests.
- BH-adjusted Significance (*p*-adj < 0.05): DEGs that have Benjamini-Hochberg (BH) adjusted *p*-values (Benjamini and Hochberg, 1995; Benjamini et al., 2009) below 0.05 are recognized as BH-significant. This adjustment method controls the false discovery rate (FDR), providing a balanced approach to identifying true positives while minimizing false positives.
- Bonferroni-corrected Significance: DEGs are considered Bonferroni-significant (Dunn, 1961) when their *p*-values remain below the threshold adjusted for the number of comparisons (0.05 divided by n, where n is the total number of genes). This stringent criterion is designed to rigorously reduce the chance of Type I errors.

Our analytical strategy for RNA-Seq data begins with a critical preprocessing step, TMM normalization. This method corrects for library size differences and compositional variations across samples, which is essential for accurate downstream analysis. We structure the count data into a DGEList object, ensuring each count is associated with its corresponding experimental condition, such as mock-infected or virus-infected OTEs at specific time points. The normalization of this data through the TMM method is the first vital step, setting the stage for a fair comparison of gene expression levels.

RNA-Seq data, characterized by count-based measurements, presents unique analytical challenges that necessitate specialized statistical treatment. One fundamental issue is the non-normal distribution of count data, which is often overdispersed, meaning the variance exceeds the mean (Law et al., 2014). Traditional parametric tests, like the two independent sample t-tests, assume normality of data and homogeneity of variances, conditions that RNA-Seq data rarely satisfy.

To address these challenges, we employed Generalized Linear Models (GLM) (Nelder and Wedderburn, 1972) to model the RNA-Seq count data. GLMs are a flexible extension of ordinary linear models that allow for response variables to have error distribution models other than a normal distribution. They are particularly suited for modeling count data that follow distributions from the exponential family, such as Poisson or negative binomial, which are adept at handling the discrete and often overdispersed nature of RNA-Seq data.

Moreover, the Quasi-Likelihood *F*-test (Wedderburn, 1974), utilized within the GLM framework, provides a robust tool for assessing the significance of differences in gene expression between experimental conditions while accommodating the inherent overdispersion. This test does not require the strict assumptions of parametric tests and instead estimates the variance and mean relationship directly from the data, allowing for more accurate and reliable inference even with complex RNA-Seq datasets.

The application of the GLM with QL *F*-test is therefore justified as it provides a methodologically sound approach to identifying differentially expressed genes in RNA-Seq data, capturing the complex distributional characteristics of such data and managing the gene-specific variability in a way that traditional methods cannot.

#### 2.1.1 Model fitting process in GLM with quasi-likelihood *F*-tests

The Generalized Linear Models with Quasi-Likelihood F-test (GLMQL) approach, as implemented in the EdgeR software package (Robinson et al., 2010), is central to this analysis and follows the following steps:

1. Design Matrix Formation:

(a) The design matrix is a critical component that represents the experimental setup, encapsulating factors that might influence gene expression, such as infection type (e.g., IAV, MPV, PIV3) and time points post-infection (e.g., 24 or 72 h).
(b) Each row in the design matrix corresponds to a sample, and each column represents a factor that may affect expression levels.

2. Fitting the GLM:

(a) With the design matrix in place, a GLM is fit (glmQLFit; Chen et al., 2020) to the TMM-normalized count data.
(b) The GLM links the expected value of the counts to the linear predictors via a link function appropriate for count data, usually the log link for Poisson or negative binomial distributions.
(c) The coefficients in the GLM represent the log fold-changes (logFC) for each factor in the design matrix when compared to a reference level, often the baseline condition such as the mock-infected samples at 24 h.

3. Quasi-Likelihood *F*-test:

(a) After model fitting, the Quasi-Likelihood F-test is used to compare the full model (with all coefficients) against a reduced model (without the coefficients of interest) to determine if the omitted factors significantly affect gene expression.
(b) This test estimates the variability (dispersion) directly from the data, which accounts for the overdispersion often seen in RNA-Seq data.
(c) It returns *p*-values indicating the probability of observing the data if the null hypothesis (no difference in expression due to the factor) were true.

4. Significance and Log Fold-Change (logFC) Extraction:

(a) For genes where the full model significantly deviates from the reduced model, the Quasi-Likelihood *F*-test provides *p*-values, which are then adjusted for multiple testing to control the false discovery rate.
(b) Simultaneously, the GLM yields logFC values, which represent the factor‘s effect size on gene expression. The logFC is the natural logarithm of the ratio of the expression level in the treatment condition to that in the reference condition.

A low *p*-value indicates strong evidence against the null hypothesis, suggesting that the factor in question does have a significant effect on gene expression. The logFC reflects how much the expression level changes due to the factor. A positive logFC indicates upregulation, while a negative logFC suggests downregulation due to the factor‘s presence.

#### 2.1.2 Biomarker identification through differential expression analysis

The culmination of our statistical methodology involves identifying potential biomarkers that can inform on the specific viral infection and its impact on gene expression. Post-application of the Generalized Linear Models with Quasi-Likelihood F-test (GLMQL), our analysis proceeds to pinpoint genes exhibiting significant differential expression between the conditions compared, using both raw *p*-values and adjusted significance levels.

We utilize the GLMQL framework for precise identification of DE genes across experimental conditions, emphasizing those with significant *p*-values and notable log fold changes (logFC). This meticulous approach facilitates the discovery of genes whose expression profiles are distinctively altered in response to viral infections, earmarking them as potential biomarkers.

Recognizing the importance of minimizing false discoveries in high-throughput data analysis, we apply rigorous multiple testing corrections. This includes the Benjamini-Hochberg (BH) method to control the false discovery rate and the Bonferroni correction for a stringent significance threshold. Such adjustments ensure that identified DE genes are not artifacts of multiple comparisons but reflect genuine biological differences.

In our analysis, we leverage the Magnitude-Altitude Score (MAS) and Relaxed Magnitude-Altitude Score (RMAS) algorithms to prioritize genes for their potential as infection-specific biomarkers. These methodologies transcend conventional prioritization based solely on *p*-values or log fold changes (logFC), as commonly seen in tools like EdgeR (Robinson et al., 2010) or DESeq2 (Love et al., 2014).

##### 2.1.2.1 MAS definition

The MAS integrates both the magnitude of expression change ( $\left|\log_{2}\left({FC}_{l}\right)\right|$ ), and its statistical significance ( $\left|\log_{10}\left(p_{l}^{BH}\right)\right|$ ) into a singular score for each significant gene l, formulated as $MAS_{l}={\left|\log_{2}\left({FC}_{l}\right)\right|}^{M}{\left|\log_{10}\left(p_{l}^{BH}\right)\right|}^{A}$ , where $p_{l}^{BH}$ denotes Benjamini-Hochberg adjusted p-values. Here M and A are hyperparameters optimizing the balance between expression change and statistical confidence, ensuring a comprehensive evaluation of each gene‘s relevance.

##### 2.1.2.2 RMAS definition

For situations where traditional FDR corrections, such as Benjamini-Hochberg, render no genes as significantly differentially expressed, the RMAS method provides an alternative. It employs *p*-values ( $p_{l})$ in place of BH-adjusted ones ( $p_{l}^{BH})$ , defined as ${\mathrm{RMAS}}_{l}=\;{\left|\log_{2}\left({FC}_{l}\right)\right|}^{M}{\left|\log_{10}\left(p_{l}\right)\right|}^{A}\text{.}$ This adjustment broadens the analysis to include genes that might be biologically significant but are overlooked due to stringent statistical corrections.

By setting M and A to 1, we ensure that the magnitude of expression change and its statistical significance are equally important in the final score. This reflects our hypothesis that genes critical to infection response should not only show significant expression changes but also be statistically robust, regardless of the magnitude of their change.

The use of MAS and RMAS offers a nuanced approach to gene prioritization by capturing a gene’s overall impact on the study’s biological context. This is particularly crucial in RNA-Seq data analysis for several reasons:

- Holistic Gene Evaluation: Traditional prioritization based on *p*-values or logFC alone might overlook genes that, despite having moderate changes in expression or borderline statistical significance, play crucial roles in biological processes or pathways. MAS and RMAS integrate both dimensions, offering a more rounded assessment of each gene‘s potential impact.
- Enhanced Biological Relevance: By balancing the magnitude of change with statistical significance, MAS and RMAS help identify genes that are not only statistically significant but also biologically meaningful. This is vital for understanding complex biological responses, such as those seen in viral infections, where the interaction between host and pathogen can affect gene expression in nuanced ways.
- Adaptability to Data Variability: RNA-Seq datasets are characterized by inherent variability and complexity. The flexibility of MAS and RMAS in considering both expression change and significance level makes them particularly suited for such data, enabling the identification of relevant genes across a range of experimental conditions.
- Exploratory Insight: RMAS, with its use of raw *p*-values, allows for the exploration of data beyond the constraints of traditional statistical thresholds. This exploratory nature is invaluable for uncovering potential biomarkers or therapeutic targets that might be dismissed by more conservative methods.

#### 2.1.3 Gene ontology analysis

To interpret the biological implications of the significant changes in gene expression observed upon viral infection, we employed Gene Ontology (GO) enrichment analysis. This analysis facilitates the understanding of the biological processes, cellular components, and molecular functions enriched among the list of differentially expressed genes, thereby offering insights into the host’s defense mechanisms and the potential strategies employed by viruses to evade these defenses.

For the GO enrichment analysis, we utilized the Enrichr platform (Chen et al., 2013), a comprehensive web-based tool designed to analyze gene lists for enrichment of specific GO terms. Enrichr incorporates multiple gene set libraries and employs robust statistical methods to identify significantly enriched terms, providing a deeper understanding of the biological themes associated with the gene lists.

Each list of significant upregulated and downregulated genes (raw *p*-values) identified for IAV, MPV, and PIV3 at 24- and 72-h post-infection was separately analyzed using Enrichr. The analysis process entailed the submission of gene symbols to the Enrichr platform, where the enrichment of GO terms across three main categories, Biological Process, Cellular Component, and Molecular Function, was assessed. The output from Enrichr included lists of enriched GO terms associated with each gene list, along with statistical metrics such as *p*-values and combined scores, which facilitated the prioritization and interpretation of the most relevant biological processes impacted by viral infection.

Through this systematic GO enrichment analysis, we aimed to identify and compare the host cellular processes modulated in response to infection by each virus, thereby shedding light on the complexity of host-pathogen interactions and the dynamic nature of the host defense mechanisms. This methodological approach not only allowed for the exploration of the specific effects of individual viruses on the host cellular landscape but also provided a framework for identifying commonalities and differences in the host responses to these respiratory viral pathogens.

### 2.2 Classifying respiratory virus infections in OTEs using GLMQL-RMAS

Our classification approach commenced with the application of the GLMQL-RMAS method to identify top genes by comparing IAV-infected OTEs against Mock samples at 24 h post-infection. Similar comparative analyses were conducted for MPV and PIV3-infected samples against Mock samples at the same time point. It is important to note that the top gene with the highest RMAS score is assigned a rank of 1, thereby assigning an RMAS rank to each gene. This initial phase allowed us to identify significant genes uniquely expressed in response to each viral infection. We further refined our analysis by identifying common significant genes across these contrasts. For these common significant genes, an aggregated RMAS rank was defined as the summation of RMAS ranks for each comparison. Utilizing the RMAS scores, the gene with the smallest aggregated RMAS rank was selected as the fingerprint marker, capable of distinguishing all infected samples from the mock samples at the 24-h post-infection mark. This procedure was replicated for samples collected at 72 h post-infection, yielding two pivotal genes indicative of infection at 24- and 72-h post-infection intervals.

In instances where the top-selected gene at 24- and 72-h post-infection are the same, we opt for the second-highest ranked gene at 24 h as the primary selection. This adjustment is made because, within the first 24 h post-infection, the samples are less separable due to the nature of the viruses, and selecting an alternate top gene enhances the separation of samples during this critical early phase. This strategy ensures a more robust marker is utilized for distinguishing samples in the initial post-infection period.

To elucidate the dynamic changes between the two time points, we applied the GLMQL-RMAS method again, comparing the gene expressions of Mock, IAV, MPV, and PIV3 infected samples at 72 h against their respective 24-h expressions. An aggregated RMAS rank for all common significant genes was then defined as the summation of ranks from all four contrasts. Subsequently, the gene with the smallest aggregated RMAS rank, capable of separating samples at times 24 and 72, was chosen as the third pivotal gene.

Following this, a log-transformation was applied to the expression data of the three selected genes to normalize the distribution of expression levels, thereby enhancing the comparability and interpretability of gene expression across samples. To classify the samples into eight distinct classes (three viruses at two time points, along with two mock conditions), we employed a multinomial logistic regression model. This model was selected for its ability to handle multiple classes and provide probabilistic insights into class membership.

We adopted a Stratified K-Fold cross-validation strategy (Rodriguez et al., 2009) with six splits to evaluate the model’s performance. This approach ensured that each fold accurately represented the entire dataset by maintaining the proportion of samples for each class, thus allowing us to gauge the model’s generalizability and robustness across different data subsets. The model’s classification performance was quantitatively assessed using metrics such as accuracy, precision, recall, and the F1-score (Theodoridis and Koutroumbas, 2006). Furthermore, an aggregated confusion matrix provided a comprehensive visual summary of the model’s performance across all folds, detailing true positives, false positives, and misclassifications.

To evaluate the efficacy of RMAS relative to ranking methods commonly utilized in EdgeR (Robinson et al., 2010) and DESeq2 (Love et al., 2014), we replicated the aforementioned process, substituting RMAS with two alternative ranking criteria: once based on the smallest *p*-value and once based on the largest log2-fold change (log2FC). This comparative analysis allowed us to assess the performance and discriminative power of RMAS against traditional approaches in identifying pivotal genes indicative of viral infection.

## 3 Results

In this section, we present the outcomes derived from the methodologies outlined in Section 2.

### 3.1 Statistical methodology and gene ontology analysis

**Figure 1:**
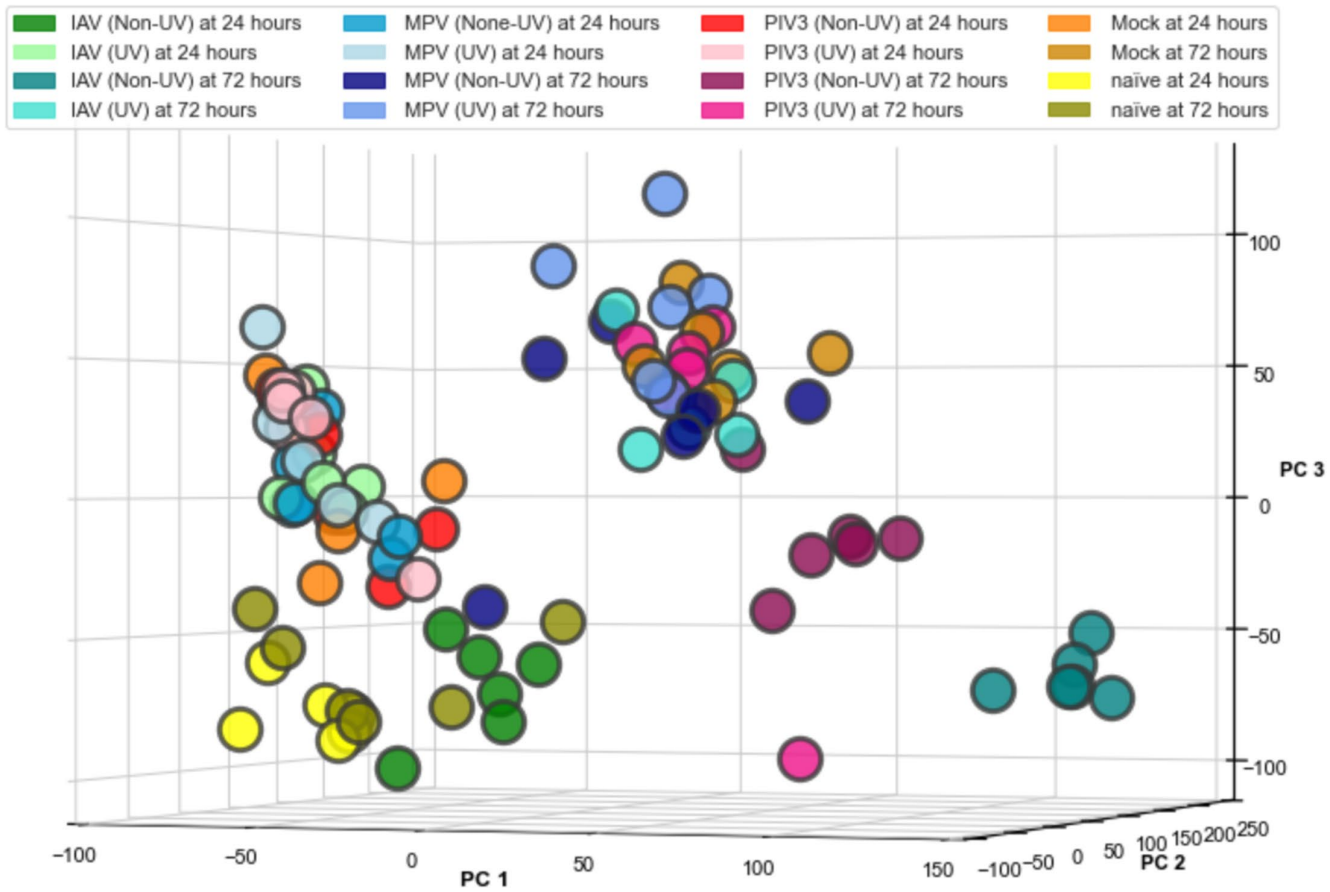
Three-dimensional principal component analysis (PCA) of RNA-Seq data from OTE samples. The PCA plot visualizes transcriptional profiles across 16 infection conditions, demonstrating distinct clustering by virus type (IAV, MPV, PIV3), treatment (UV-treated, Non-UV/active), and time post-infection (24, 72 h), including Mock and Naïve controls at both time points. Data points represent individual samples, color-coded by condition, as listed in Table 1. The log2 transformation was applied solely for the purpose of this visualization to standardize variance; subsequent analyses were conducted with non-transformed data.

For the purpose of data visualization in the PCA, we applied a log2-transformation to the RNA-Seq data post-TMM normalization, facilitating the equalization of variance across samples. Figure 1 presents the 3D PCA plot, which provides a clear spatial segregation of the samples according to the defined experimental groups from Table 1, demonstrating the distinct transcriptional profiles induced by each viral infection condition. It is important to note that the log2-transformation was specifically for PCA visualization; all other analyses reported in this paper are based on non-log-transformed data to preserve the original distribution and scale of expression values.

**Figure 2:**
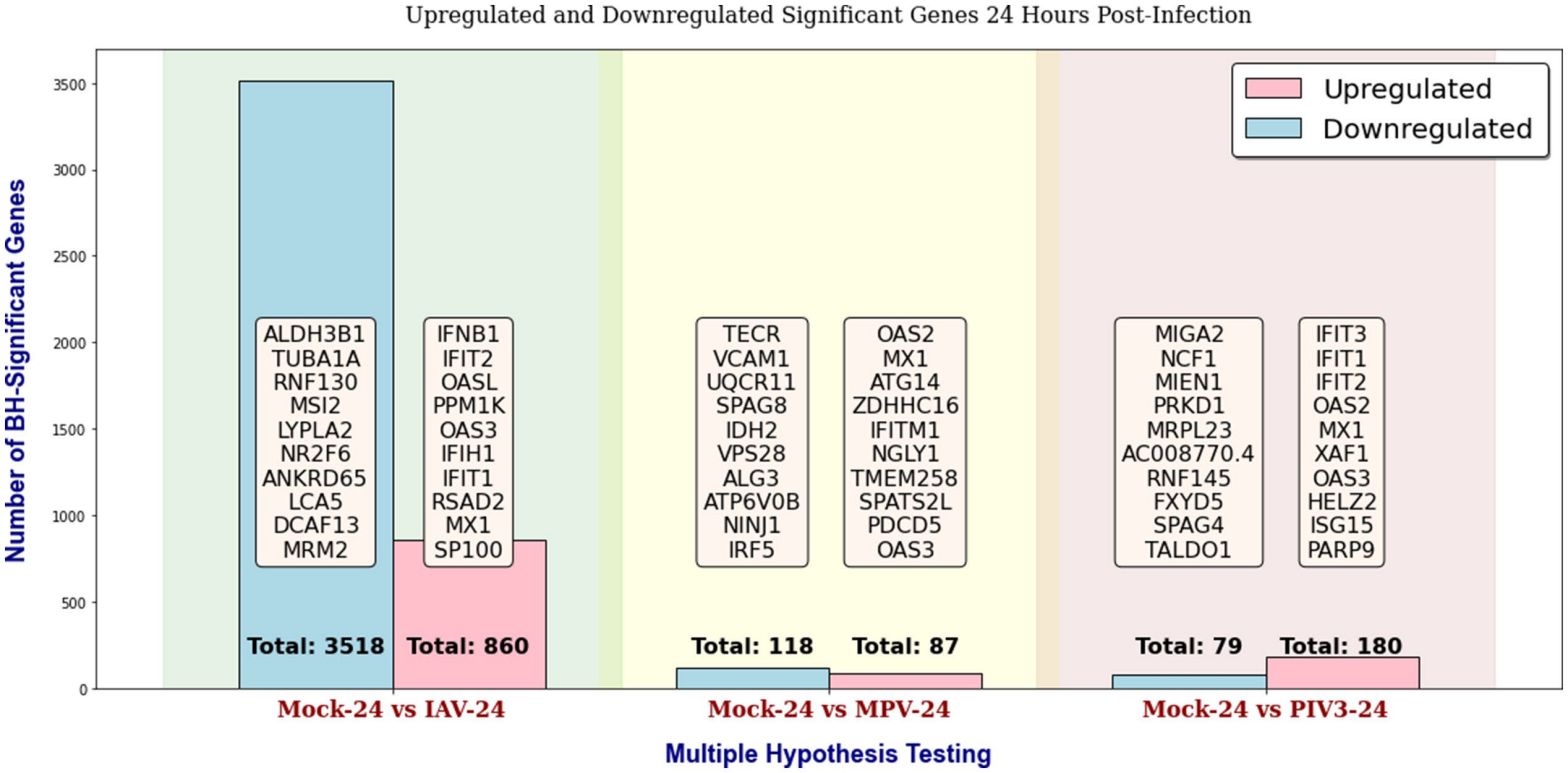
Distribution of upregulated and downregulated significant genes in IAV, MPV, and PIV3 infected samples compared to Mock samples at 24 h post-infection, using raw *p*-values with a significance level of *α* = 0.05.

**Figure 3:**
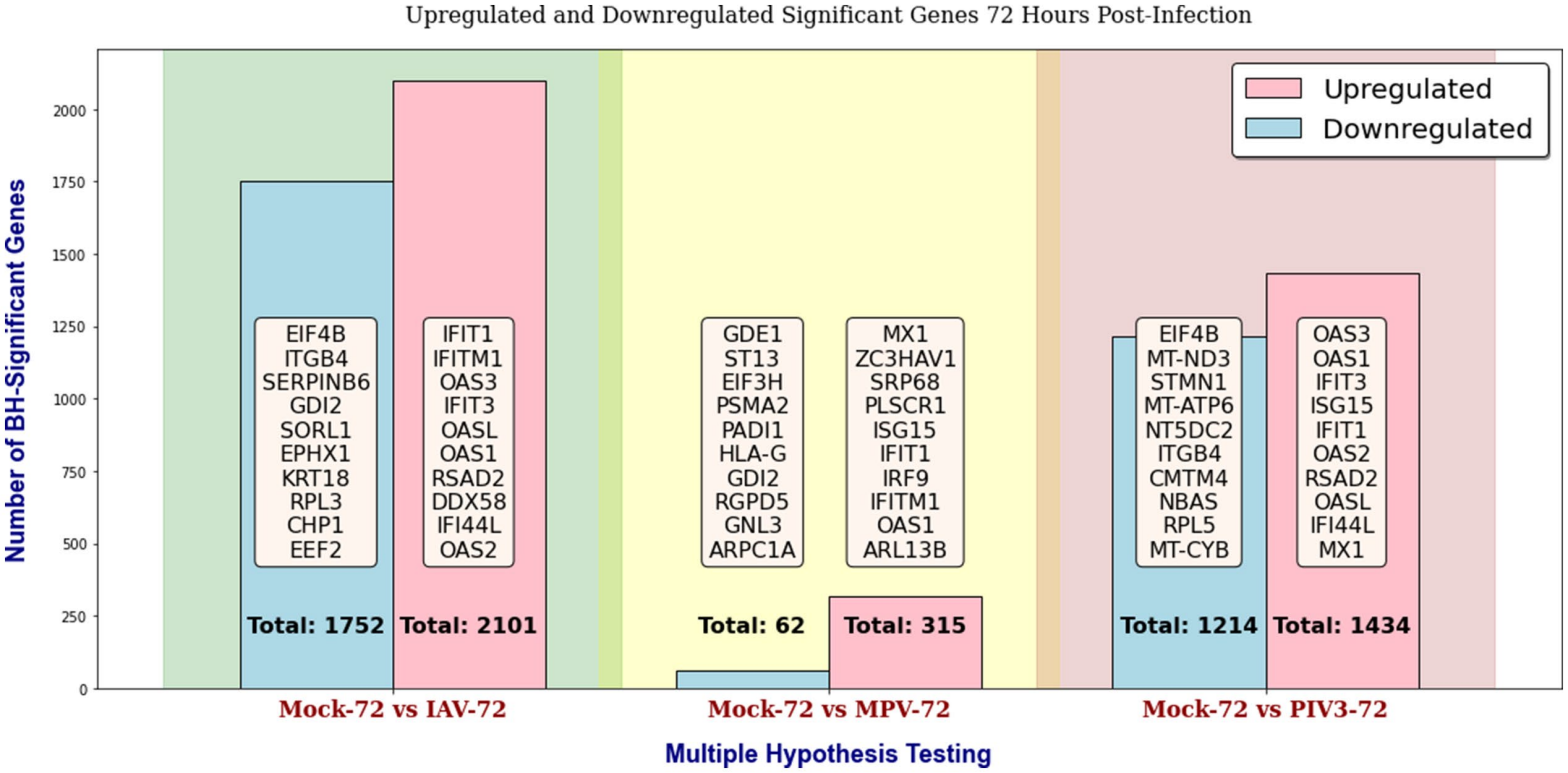
Distribution of upregulated and downregulated significant genes in IAV, MPV, and PIV3 infected samples compared to Mock samples at 72 h post-infection, using raw *p*-values with a significance level of *α* = 0.05.

Throughout this study, in the GLMQL-RMAS/MAS analysis, we set both the M and A parameters to 1, with a significance level of *α* = 0.05. Figures 2, 3 display the total counts of upregulated and downregulated significant genes in IAV, MPV, and PIV3 infected samples, contrasted against Mock samples at 24- and 72-h post-infection, respectively. Supplementary Figure S1 displays volcano plots for six contrasts at both 24- and 72-h post-infection using raw *p*-values. UV-treated samples served primarily as in-house controls to ascertain the baseline effects of viral component presence without replication. For a thorough analysis, we also conducted multiple hypothesis testing across all actively infected samples compared to their UV-treated counterparts, detailed in Supplementary Figure S2.

Supplementary Figures S3, S4 display the total counts of upregulated and downregulated genes, significant according to both BH and Bonferroni corrections, in samples infected with IAV, MPV, and PIV3, contrasted against Mock samples at 24- and 72-h post-infection, respectively. After applying the Benjamini-Hochberg adjustment and Bonferroni correction, all MPV-associated genes were rendered insignificant, attributed to the nature of the MPV virus. Consequently, for the remainder of this paper, we will focus our discussion on significant genes identified using raw p-values, as shown in Figures 2, 3.

**Figure 4:**
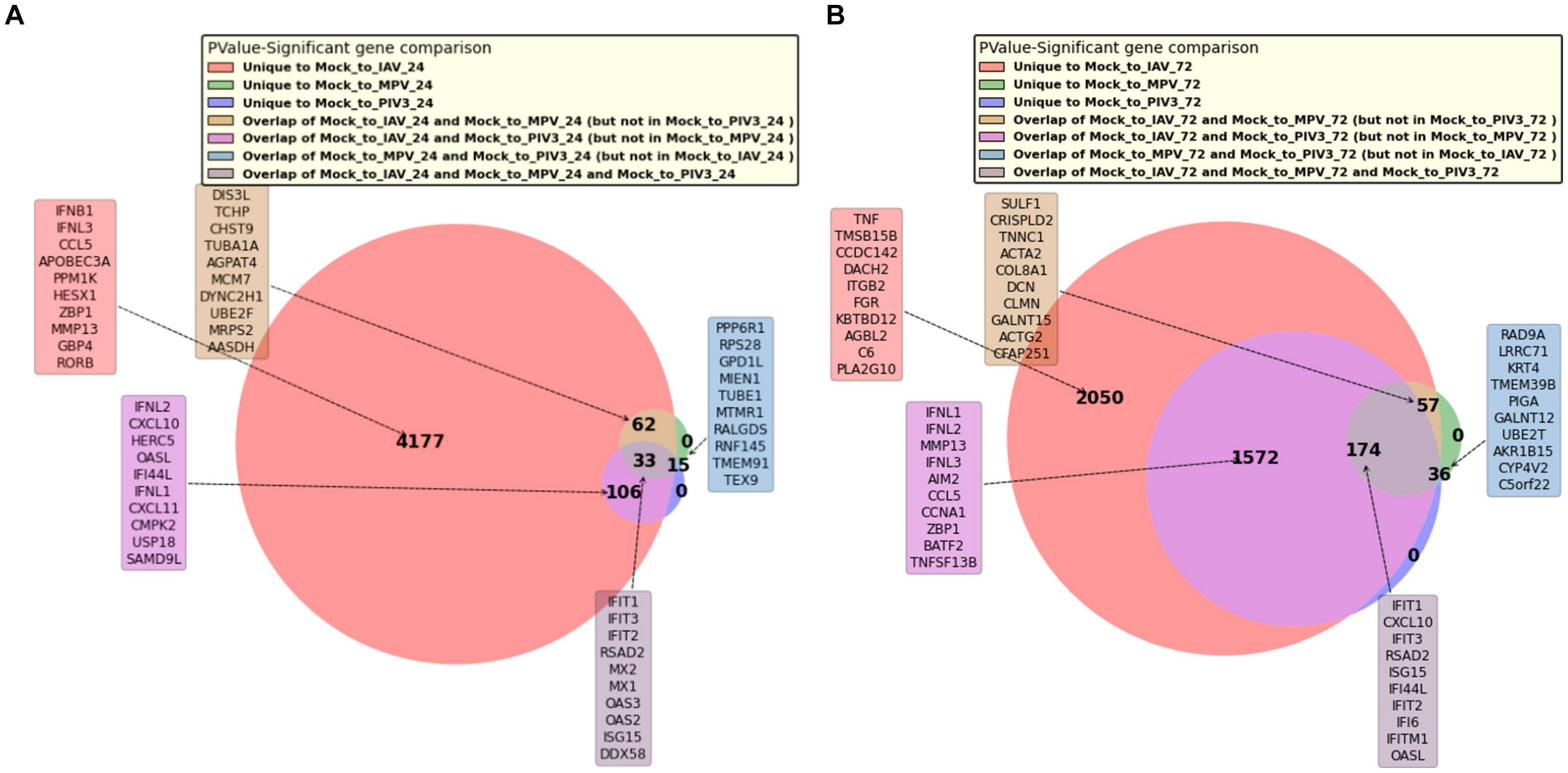
Venn diagrams showing the overlap of significant genes associated with IAV, MPV, and PIV3 infections compared to Mock samples at 24/72 h post-infection. Genes are ranked based on RMAS scores and aggregated RMAS rankings to highlight their significance across the groups. **(A)** At 24 h post-infection. **(B)** At 72 h post-infection.

**Figure 5:**
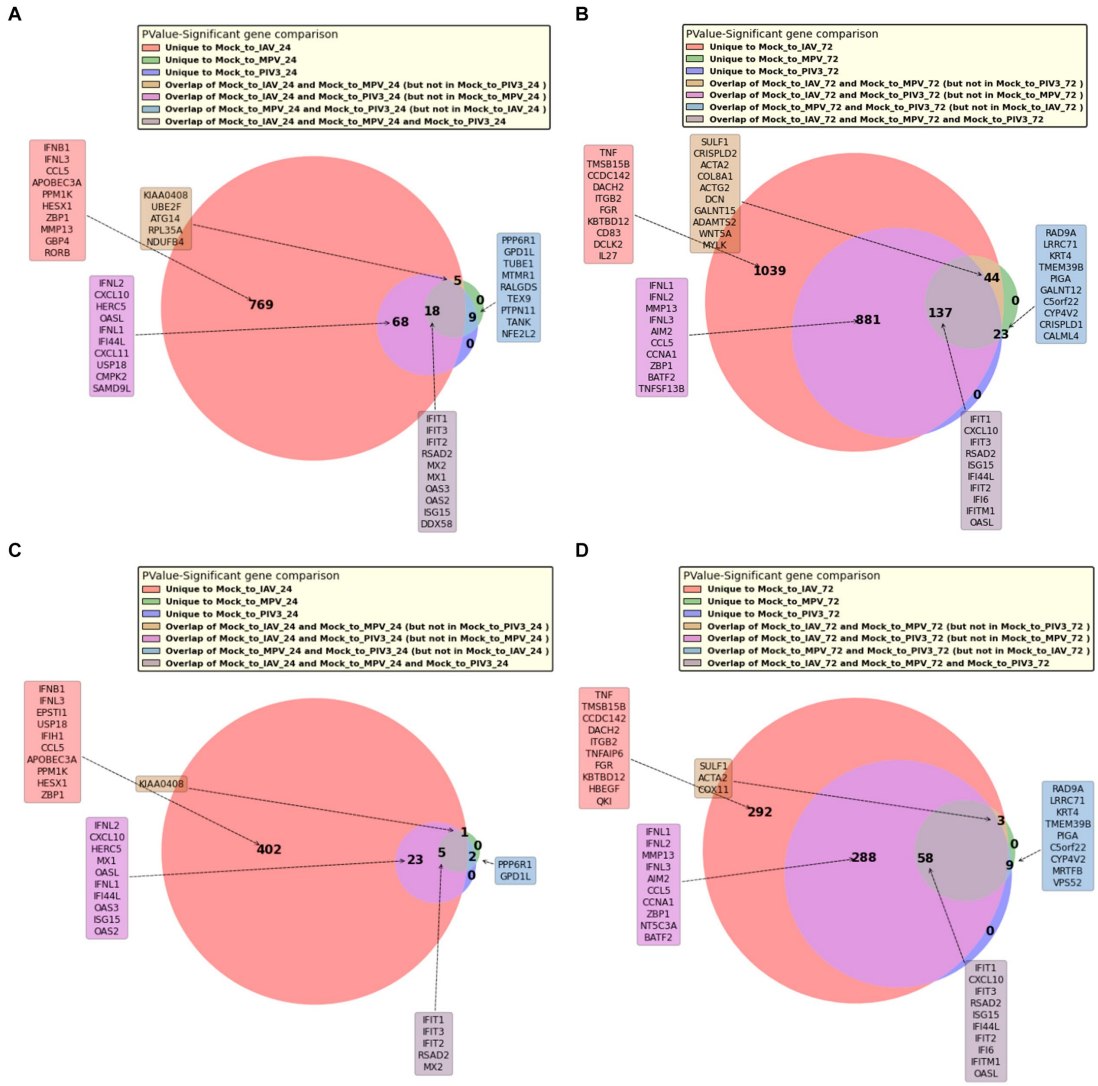
Venn diagram of significant upregulated genes with logFC >1 at 72 h post-infection. Consistent with the previous figures, it demonstrates the dependability of RMAS ranking in maintaining the identification of the top gene despite varying logFC thresholds. **(A)** Venn diagram of significant upregulated genes with logFC >0 at 24 h post-infection. **(B)** Venn diagram of significant upregulated genes with logFC > 0 at 72 h post-infection. **(C)** Venn diagram of significant upregulated genes with logFC > 1 at 24 h post-infection. **(D)** Venn diagram of significant upregulated genes with logFC > 1 at 72 h post-infection.

Figures 4A,B present Venn diagrams illustrating the common significant genes associated with IAV, MPV, and PIV3, contrasted against Mock samples at 24- and 72-h post-infection, respectively. Supplementary Figures S5a–d display Venn diagrams showcasing the common BH and Bonferroni significant genes related to IAV, MPV, and PIV3 when compared against Mock samples at the same post-infection intervals. The genes are ranked according to their RMAS scores and aggregated RMAS rankings, reflecting their overall significance across two or three groups. Figures 5A–D displays the Venn diagrams for significant upregulated genes when logFC >0 and logFC >1. This figure aims to demonstrate the stability of RMAS ranking, showing that it remains consistent regardless of the logFC lower threshold (with the top gene being identically selected), unlike rankings based on only p-values.

#### 3.1.1 Gene ontology analysis

The GO analysis aimed to decipher the complex interactions between viral infections and host cellular mechanisms, focusing on the biological processes impacted by the upregulation and downregulation of significant genes.

At 24 h post-infection, GO analysis (Supplementary Tables S1, S3, S5) revealed an upregulation of genes associated with innate immune responses and antiviral defense mechanisms across all three viral infections, indicating a robust host defense strategy that involves interferon-stimulated genes and cytokine signaling pathways. Concurrently, the downregulation of genes (Supplementary Tables S2, S4, S6) highlighted the viruses’ impact on host cellular structures, metabolism, and organelle functions, suggesting viral strategies to disrupt normal cell processes and evade immune surveillance.

By 72 h post-infection, the host response showed a sustained activation of antiviral defense mechanisms (Supplementary Tables S7, S9, S11), with a continued focus on cytokine-mediated immune responses and the inhibition of viral replication. This period also exhibited a significant downregulation in genes related to translation, cellular respiration, and gene expression (Supplementary Tables S8, S10, S12), reflecting a deeper viral influence on host metabolic and biosynthetic pathways.

Supplementary Tables S13, S14 provided a comparative analysis of the common host responses to IAV, MPV, and PIV3 infections at both time points. These analyses underscored a conserved set of defense mechanisms activated by the host, including the upregulation of genes crucial for blocking viral entry, replication, and the modulation of immune signaling. This shared response highlights the fundamental aspects of the host’s antiviral defense and points to potential targets for broad-spectrum therapeutic interventions.

### 3.2 Classifying respiratory virus infections in OTEs using GLMQL-RMAS

**Figure 6:**
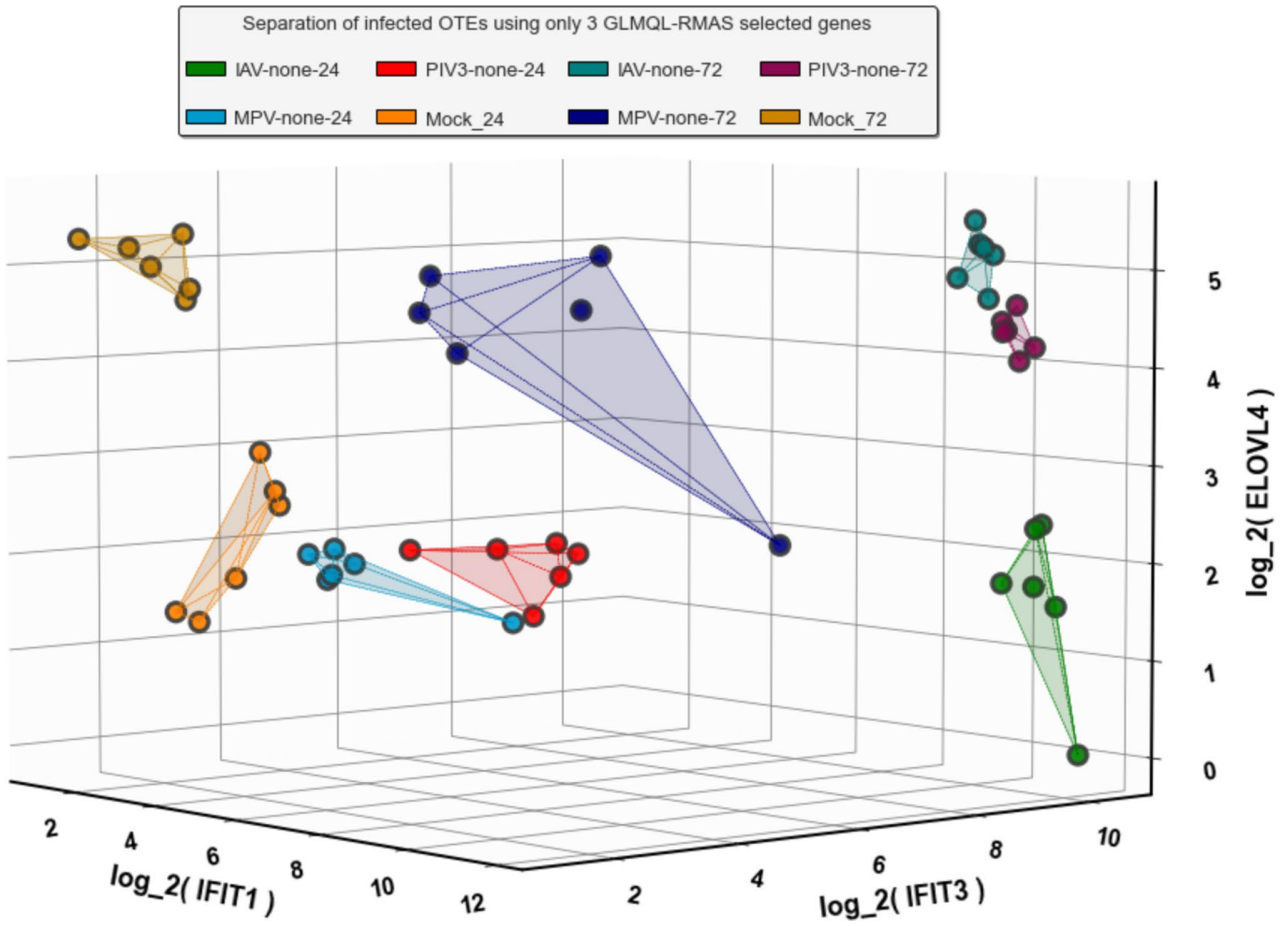
Three-dimensional visualization of sample classification using IFIT1, IFIT2, and ELOVL4 as axes. This representation underscores the distinct clustering of samples based on infection and time-dependent gene expression patterns, facilitated by the GLMQL-RMAS methodology.

**Figure 7:**
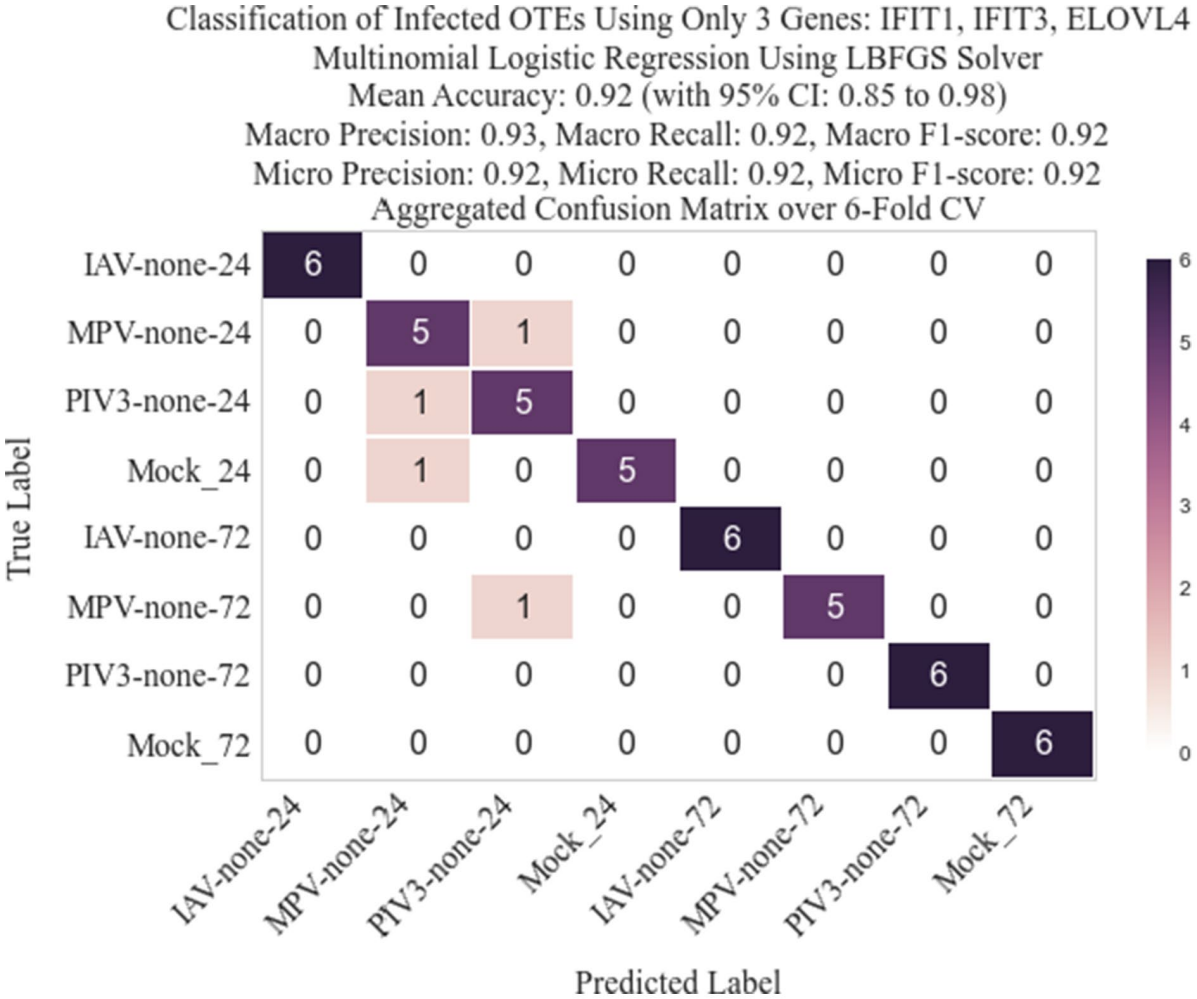
Confusion matrix for the 8-class classification using IFIT1, IFIT2, and ELOVL4 as predictors. The matrix highlights the model’s accuracy in distinguishing between different viral infections and time points, reflecting a mean accuracy of 92% as achieved through a 6-fold stratified cross-validation process.

**Figure 8:**
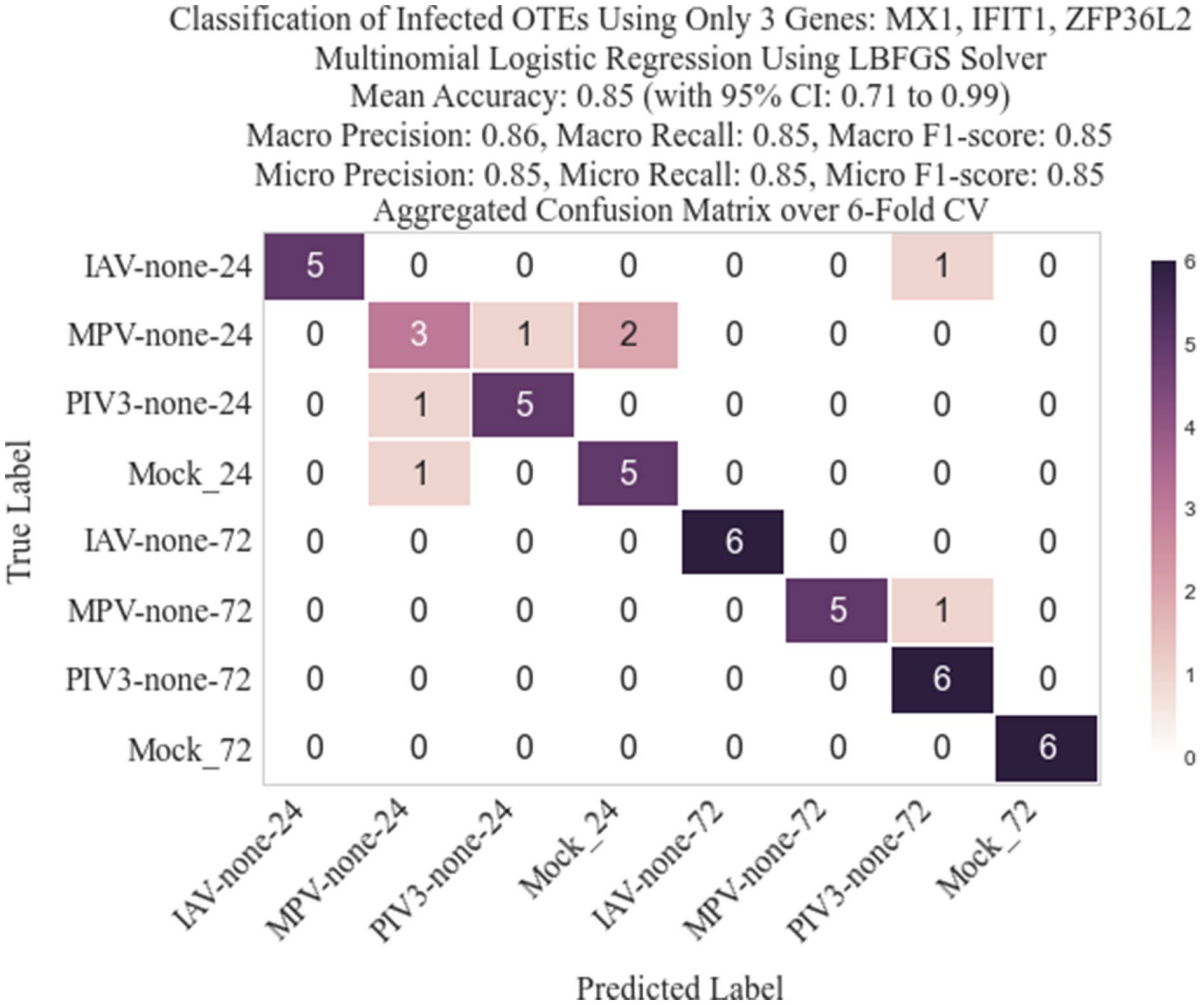
Confusion matrix for the 8-class classification after replacing RMAS ranking with rankings based on the smallest *p*-value (EdgeR ranking method).

**Figure 9:**
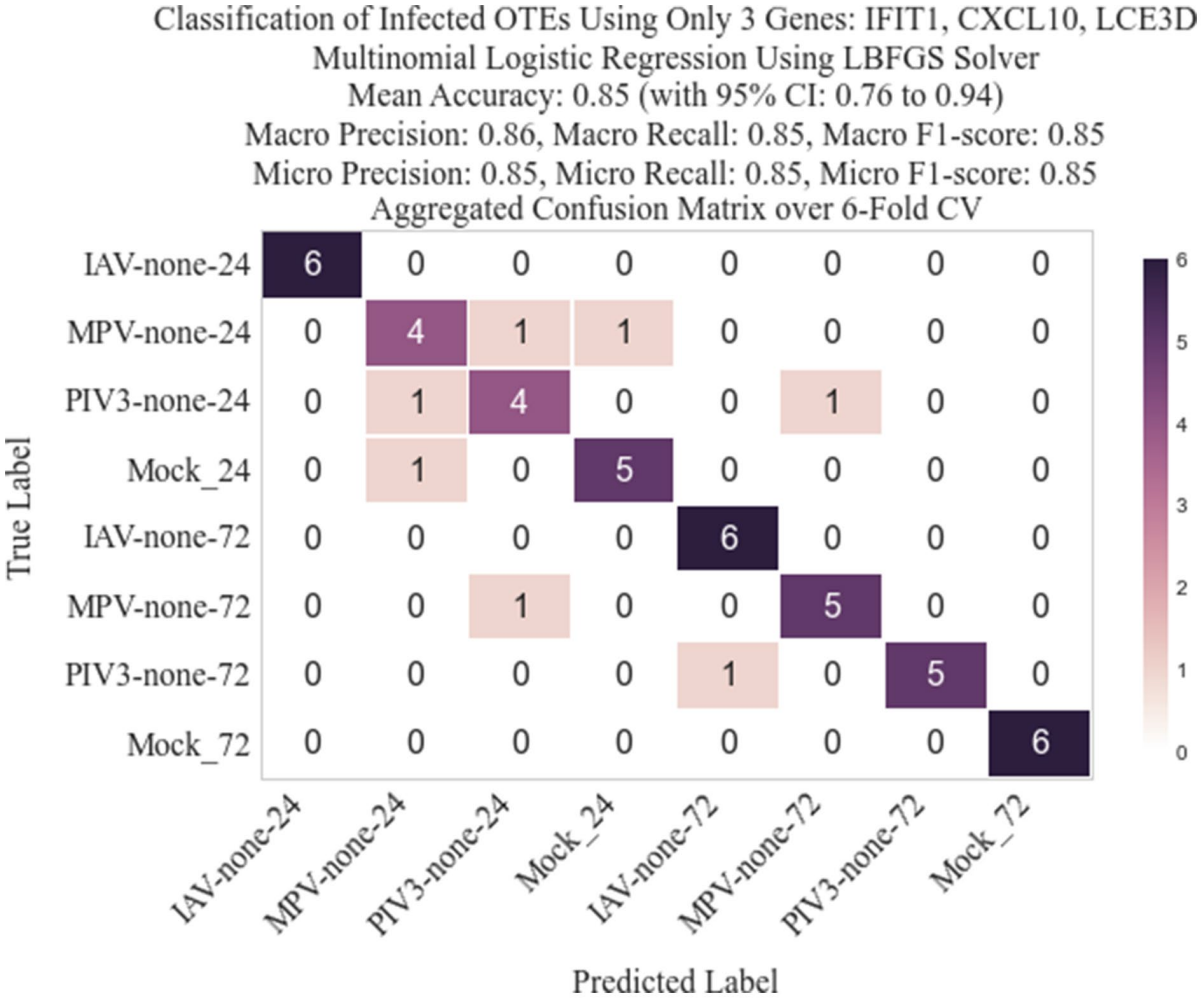
Confusion matrix for the 8-class classification when genes are ranked based on logFC (EdgeR ranking method).

Following the methodology described in Section 2.3, we identified three genes: IFIT1 (infection-dependent), IFIT2 (infection-dependent), and ELOVL4 (time-dependent), using GLMQL-RMAS as predictors for multinomial logistic regression. After conducting a 6-fold stratified cross-validation, we obtained a mean accuracy of 92% with a 95% confidence interval of [85, 98%]. Figure 6 displays a 3D visualization of all samples using only these three genes as the coordinate axes. Figure 7 shows the confusion matrix for the 8-class classification using these genes. Figure 8 presents the confusion matrix for the 8-class classification when replacing RMAS with the common ranking method, ranking genes based on the smallest *p*-value, and Figure 9 illustrates the classification when genes are ranked based on logFC, with all other aspects remaining identical.

## 4 Discussion

### 4.1 Statistical methodology and gene ontology analysis

The PCA visualization (Figure 1) reveals that samples progressively shift towards the positive direction of PC1 from 24 to 72 h post-infection. Figures 2, 3 and Supplementary Figures S3, S4 suggest the stability of the MAS/RMAS ranking method, as it maintains consistent rankings even when applying Benjamini-Hochberg or Bonferroni corrections, provided the genes still meet the significance criteria. Notably, IFNB1 and IFIT1 remain the top-ranked upregulated significant genes when contrasting IAV samples against Mock samples at both 24- and 72-h post-infection. This stability in ranking is further highlighted in Figure 5, where using RMAS for ranking shows that even when adjusting the lower logFC threshold from 0 to 1, the ranking of upregulated significant genes remains unchanged.

Analyzing the upregulated genes at 24 h post-infection (Figure 2), we observe a distinct immunological response pattern for each virus:

- For IAV, genes such as IFNB1 (interferon-beta 1) and IFIT2 (interferon-induced protein with tetratricopeptide repeats 2) are central to the innate antiviral response. IFNB1 is pivotal for initiating a broad-spectrum antiviral state, while IFIT2 is known for its role in inhibiting viral protein synthesis. OASL (2′-5′-oligoadenylate synthetase-like) and RSAD2 (radical S-adenosyl methionine domain-containing 2) enhance viral RNA degradation, indicating a robust cellular mechanism to thwart viral replication.
- For MPV, OAS2 and MX1 (myxovirus resistance 1) suggest activation of similar antiviral pathways. MX1 is involved in the inhibition of viral replication in the cytoplasm, adding an additional layer of cellular defense. ATG14 and ZDHHC16 (zinc finger DHHC-type containing 16) may indicate the engagement of autophagy-related pathways and membrane trafficking adjustments as cellular strategies against viral infection.
- In the case of PIV3, IFIT3 (interferon-induced protein with tetratricopeptide repeats 3) upregulation is significant for the interception of viral replication, complementing the activities of IFIT1 and IFIT2. The increase in OAS2 and MX1 once again underscores a shared antiviral strategy across different viral infections, and ISG15 (interferon-stimulated gene 15) points to a broader modulation of the immune response, given its role in protein ubiquitination related to antiviral defense.

At the 72-h mark (Figure 3), the trend of gene expression changes suggests an adaptive shift in the host response. For instance, the upregulation of IFITM1 and OAS1 in IAV infections might signify a sustained defense mechanism, possibly transitioning from an immediate to a more regulated long-term response. The continued downregulation of structural genes like ITGB4 and metabolic genes like EEF2 across different viruses could reflect a sustained reprogramming of cellular processes in the extended phase of viral infection. At 72 h post-infection, there is a clear pattern of upregulation for genes involved in the OTE’s response to viral infection:

- For IAV, we see a sustained upregulation of IFIT1 and IFITM1, which are critical for the ongoing defense against viral replication and signaling to other immune system components. OAS3, OASL, and OAS1 continue to illustrate the activation of the oligoadenylate synthetase pathway, crucial for degrading viral RNA. RSAD2 and DDX58 (RIG-I) remain central for recognizing RNA viruses and initiating immune responses, suggesting a prolonged active defense mechanism.
- For MPV, the persistence of MX1 and OAS1 upregulation is noted, suggesting a long-lasting immune response activation. Proteins like ZC3HAV1 (zinc-finger antiviral protein) play roles in viral mRNA sensing and decay, indicating a continued cellular effort to suppress viral gene expression. SRP68 and PLSCR1 implicate ongoing cellular adjustments in response to infection stress.
- The PIV3 response highlights a similar trend with OAS3 and OAS1 again pointing to the significance of the antiviral state at this later time point. IFIT3, ISG15, and IFI44L emphasize a maintained immune response, with ISG15 indicating a broader immune system communication and potential modulation of the inflammatory response.

In our extensive investigation of the host response to IAV, MPV, and PIV3 infections in OTEs at 24- and 72-h post-infection, Gene Ontology (GO) analysis has been instrumental in elucidating the complex interactions between viral invasion and host defense mechanisms. By analyzing both upregulated and downregulated genes at these time points, we have painted a detailed portrait of how host cells activate various biological processes to counteract viral threats. Concurrently, these cells undergo substantial changes in cellular functions to support the viral lifecycle. Given the absence of BH or Bonferroni significant genes for the MPV virus, we conducted the GO analysis using significant genes determined by raw *p*-values. This approach ensured a comprehensive understanding of the host’s response mechanisms across all studied viruses.

#### 4.1.1 Gene ontology analysis of the host (OTE) response to influenza A virus

Our examination of OTEs infected with IAV at 24 h post-infection through Gene Ontology (GO) analysis (Supplementary Table S1) has provided deep insights into the host’s defense mechanisms. This analysis reveals a multifaceted response to viral invasion, highlighting the significant roles played by a diverse array of genes. Key among these are interferon-stimulated genes such as IFITM3, IFITM1, IFITM2, and OAS1, which spearhead the defense against viral entry, replication, and spread. Additionally, genes integral to the innate immune response, such as CGAS and DDX60, have been identified, emphasizing the host’s rapid mobilization against the virus.

The breadth of the host’s defense is further illustrated through the “Defense response to symbiont” category, pointing to a strategy effective against a range of pathogens. This broad-based approach is complemented by more targeted responses, such as those in the “Negative regulation of viral process” and “Negative regulation of viral genome replication” categories, where genes like ZC3HAV1 and APOBEC3G play pivotal roles in thwarting viral RNA degradation and replication.

The analysis also uncovers the nuanced balance the host maintains through the “Regulation of viral genome replication,” revealing the intricate dance between host and virus. The importance of cytokine signaling in coordinating the immune response is underscored by findings in “Response to cytokine” and “Response to type II interferon” categories, with genes like CD40 and GCH1 highlighting the orchestration of inflammation and antiviral states.

Moreover, the host’s adaptive response is evident in the modulation of immune and inflammatory pathways, as shown by the involvement of genes in “Positive regulation of intracellular signal transduction” and “Regulation of I-kappaB kinase/NF-kappaB signaling.” This adaptability is crucial for mounting an effective defense and is further highlighted by the host’s responsiveness to cytokine signals, as seen in the “Cellular response to cytokine stimulus” category.

On the flip side, the analysis of downregulated genes (Supplementary Table S2) reveals a significant reconfiguration of cellular priorities in the wake of IAV invasion. This includes alterations in cilium assembly and mitochondrial ribosome assembly, indicative of a strategic shift towards supporting viral replication at the expense of certain host functions.

At 72 h post-infection (Supplementary Tables S7, S8), our GO analysis traces the evolution of the host’s defense mechanisms, marking both continuity and adaptation. The sustained antiviral response, enriched in “Defense response to virus” and broadened by “Defense response to symbiont,” highlights enduring strategies against viral challenges. The ongoing efforts to curb viral RNA degradation and replication, as seen in “Negative regulation of viral process” and its counterpart for viral genome replication, reflect a persistent molecular defense.

This period also showcases the host’s regulatory finesse in “Regulation of viral genome replication,” ensuring viral control without compromising cellular integrity. The pivotal role of cytokine signaling in this extended defense phase is evident, with genes implicated in cytokine and inflammatory response modulation playing key roles in maintaining the systemic defense against IAV.

Furthermore, the activation typically associated with bacterial infections, as seen in “Cellular response to lipopolysaccharide” and “Response to lipopolysaccharide,” suggests a heightened state of immune readiness, potentially enhancing the host’s capability to manage co-infections.

The critical role of NF-kappaB signaling in mediating these responses, as highlighted in “Regulation of I-kappaB kinase/NF-kappaB signaling,” underscores the complexity and efficacy of the host’s defense mechanisms, offering insights into potential therapeutic targets to bolster resistance against IAV.

The examination of downregulated genes at this juncture sheds light on the long-term impacts of IAV on the host’s cellular machinery, revealing strategies likely aimed at optimizing viral replication and survival. This includes a notable suppression of host cell translation machinery and biosynthetic processes, suggesting a comprehensive viral strategy to reshape host cellular architecture and function.

Collectively, these insights from the GO analysis at both 24- and 72-h post-infection provide a comprehensive view of the host’s dynamic and evolving response to IAV, highlighting the complexity of antiviral defense mechanisms and identifying avenues for therapeutic intervention to support the host’s immune defense and recovery.

#### 4.1.2 Gene ontology analysis of the host (OTE) response to human metapneumovirus

Our investigation into the response of OTEs to MPV infection, at both the early and later stages post-infection (Supplementary Tables S3, S4, S9, S10), through Gene Ontology (GO) analysis, sheds light on the intricate defense mechanisms mobilized by the host. This analysis identifies key biological processes and genes that are pivotal in the host’s strategy against MPV, revealing a nuanced and adaptive response to the viral threat.

Central to the host’s defense are the “Defense response to symbiont” and “Defense response to virus” processes, which underscore a comprehensive antiviral response encompassing a wide spectrum of strategies. This is exemplified by the activation of genes such as IFITM1, RSAD2, and the OAS gene family, alongside interferon-stimulated genes (ISGs) like MX1, MX2, and ISG15. These genes are instrumental in halting viral replication and spread, highlighting the host’s rapid and targeted defense mechanisms designed to counteract MPV invasion efficiently.

The host’s efforts to thwart viral proliferation are further evidenced by the “Negative regulation of viral genome replication” and “Negative regulation of viral process,” which focus on the molecular inhibition of viral replication. This strategic suppression, involving genes like IFIT1 and PLSCR1, signifies the host’s calculated approach to limit viral dissemination. Moreover, the “Positive regulation of type I interferon production” and related categories emphasize the host’s endeavor to boost the production of critical antiviral cytokines, a vital step in fortifying the host’s antiviral state and alerting adjacent cells to the viral presence.

Additionally, the GO analysis brings to light the modulation of the immune response through specific cytokines and enzymatic activities, as seen in the “Interleukin-27-mediated signaling pathway.” This not only demonstrates the host’s readiness and sophisticated response to viral encounters but also points to potential therapeutic targets that could enhance host defenses against MPV.

Conversely, the examination of downregulated genes during MPV infection unveils viral strategies aimed at manipulating host cellular functions and immune responses. This includes interference with “Lysosomal Lumen Acidification” and “Ribosome Assembly,” potentially affecting protein degradation and synthesis. Such viral tactics may aim to divert host mechanisms to favor viral replication or impede host defenses.

Insights into the “Regulation of Neutrophil Migration” and “Regulation of Cytoplasmic Transport” reveal viral influences on immune cell dynamics and intracellular movement, possibly altering the inflammatory landscape and host protein production priorities. Furthermore, changes in “G Protein-Coupled Acetylcholine Receptor Signaling Pathway” and “Regulation of Early Endosome to Late Endosome Transport” suggest viral modifications to cellular signaling and trafficking, which could impact viral entry and immune responses.

Through this comprehensive analysis, we delve into the dynamic interactions between MPV and the host, uncovering the adaptive and multifaceted nature of the host’s defense mechanisms. These findings not only deepen our understanding of the biological processes at play during MPV infection but also offer a groundwork for developing strategies to restore host functions and bolster the immune defense, providing a clear direction for future therapeutic interventions.

#### 4.1.3 Gene ontology analysis of the host (OTE) response to parainfluenza virus type 3

Our investigation into PIV3 infection in OTEs at 24- and 72-h post-infection, documented in Supplementary Tables S5, S6, S11, S12 has yielded insightful revelations about the host’s defense strategy through Gene Ontology (GO) analysis. This detailed examination highlights a nuanced host response meticulously orchestrated to counteract PIV3 invasion, spotlighting specific biological processes and genes pivotal in mounting an effective defense.

At the forefront of this defense is the “Defense response to virus” category, which brings to light the host’s reliance on interferon-stimulated genes (ISGs) like IFITM1, IFIT5, and the OAS gene family. These genes are instrumental in blocking viral entry and replication, epitomizing the host’s immediate and robust countermeasures against the viral threat.

Moreover, the “Defense response to symbiont” term underscores the host’s broad immune capability, showcasing its adaptability to combat not just viruses but a spectrum of pathogens. This is complemented by efforts detailed under “Negative regulation of viral genome replication,” where the host employs a targeted molecular assault on the viral lifecycle, as evidenced by the actions of RSAD2 and MX1.

The analysis also shines a light on the “Antiviral innate immune response,” underscoring the innate mechanisms activated to detect and neutralize viral components, thereby initiating a comprehensive immune response. The significance of cytokine signaling in orchestrating these defenses is elaborated through the “Interleukin-27-mediated signaling pathway” and “Cytokine-mediated signaling pathway,” highlighting the crucial roles of genes that facilitate antiviral states and mobilize immune cells to the infection site.

On the flip side, the examination of downregulated genes presents a clear picture of PIV3’s strategic impact on host metabolism and cellular processes. This includes notable disruptions in carboxylic acid catabolism and the cellular response to hypoxia, indicating a viral-induced shift in host energy metabolism and adaptations to the metabolic requirements imposed by the infection.

Processes like “Carboxylic Acid Catabolic Process” and “Negative Regulation of Cellular Response to Hypoxia” point to a deliberate alteration in energy management and hypoxic responses, suggesting the virus’s influence on host cellular conditions to favor its replication. Additionally, “Response to Hydroperoxide” and related changes in the oxidative stress response highlight the host’s adjustments to manage cellular damage and foster tissue repair under viral attack.

This comprehensive analysis not only deepens our understanding of the sophisticated interplay between PIV3 and its host but also illuminates potential targets for therapeutic intervention. By highlighting the intricate defense mechanisms activated by the host and the strategic viral maneuvers to subvert these responses, we identify avenues to strengthen host defenses, aiming to mitigate the impact of PIV3 infection and support the host’s recovery and resilience against viral challenges.

#### 4.1.4 Comparative analysis of host (OTE) response to IAV, MPV, and PIV3 infections

Our extensive Gene Ontology (GO) analysis across OTEs infected with Influenza A virus (IAV), Human Metapneumovirus (MPV), and Parainfluenza virus type 3 (PIV3) at both 24 and 72 h post-infection unveils a dynamic and multifaceted host defense against these respiratory viruses. Each virus triggers a distinctive host response, leveraging a blend of common and unique strategies to counter viral threats effectively. This response encompasses a broad activation of interferon-stimulated genes, innate immune mechanisms, and a strategic modulation of cellular processes to optimize defense while navigating viral evasion tactics.

IAV elicits a potent antiviral defense, characterized by the mobilization of key interferon-stimulated genes (IFITM3, IFITM1, IFITM2, OAS1) and critical innate immune response genes (CGAS, DDX60). This robust response is augmented by a broad “Defense response to symbiont,” signifying the host’s versatile defense strategy against various pathogens. The host’s approach is further defined by targeted molecular interventions (ZC3HAV1, APOBEC3G) to curb viral proliferation, underpinned by a critical emphasis on cytokine signaling to orchestrate an integrated immune and inflammatory response, highlighting the role of genes such as CD40 and GCH1.

MPV infection showcases a similar reliance on a comprehensive antiviral response, with genes like MX1, MX2, ISG15, IFITM1, and RSAD2 playing pivotal roles in halting viral replication. This response is coupled with strategic molecular efforts (IFIT1, PLSCR1) to suppress viral replication and amplify type I interferon production, underscoring the host’s adaptive immune response modulation.

PIV3 prompts a refined host defense, focusing on obstructing viral entry and replication through the activation of IFITM1, IFIT5, and the OAS gene family. This targeted defense is complemented by a “Defense response to symbiont,” illustrating a broad immune capability. Moreover, PIV3 infection underlines the host’s direct efforts (RSAD2, MX1) to dampen viral processes and genome replication, spotlighting the role of innate immune mechanisms and cytokine signaling in mounting a coordinated defense.

#### 4.1.5 Upregulated genes and host defense mechanisms

Across all three viruses, a significant upregulation of genes involved in the “Defense response to virus” (GO:0051607) was observed at both 24 (Supplementary Tables S1, S3, S5) and 72 h (Supplementary Tables S7, S9, S11, S14) post-infection, highlighting a robust antiviral defense strategy marked by the activation of interferon-stimulated genes (ISGs) such as IFITM3, RTP4, OAS1, IFITM1, and MX1. This universal response underlines the host’s capacity to mount a strong defense, emphasizing mechanisms like viral entry blockade and innate immune signaling. Additionally, the “Negative regulation of viral process” (GO:0048525) and related terms across both time points underscore the host’s molecular strategies to restrict viral replication, featuring genes like ZC3HAV1, APOBEC3G, RSAD2, and MX1.

The cytokine response, particularly the “Response to cytokine” (GO:0034097) and “Response to type II interferon” (GO:0034341), further emphasizes the critical role of cytokine signaling in orchestrating a coordinated immune response, with genes such as STAT1 and MX2 playing pivotal roles.

#### 4.1.6 Downregulated genes and cellular alterations

Notably, the downregulation of genes associated with “Cilium Assembly” (GO:0060271) and “Organelle Assembly” (GO:0070925) at 24 h post-IAV infection (Supplementary Table S2), and similar trends in MPV (Supplementary Table S4) and PIV3 infections (Supplementary Table S6), suggests a strategic viral interference with cellular structures and organelle functions. This might facilitate viral evasion or replication by altering cellular priorities and homeostasis. Furthermore, the downregulation observed in “Translation”(GO:0006412) and “Cytoplasmic Translation” (GO:0002181) at 72 h post-IAV and PIV3 infection (Supplementary Tables S8, S12) points towards a viral strategy to dominate the host cell’s protein synthesis machinery.

#### 4.1.7 Integrated host response across viral infections

The GO analysis of genes common to IAV, MPV, and PIV3 infections at both 24 (Supplementary Table S13) and 72 h (Supplementary Table S14) post-infection reveals a core set of biological processes activated in response to these viral infections. This integrated host defense mechanism, involving both upregulation of antiviral genes and downregulation of genes related to cellular maintenance and metabolism, suggests a strategic host response that prioritizes defense mechanisms while modulating cellular functions to mitigate viral invasion.

#### 4.1.8 Comparative analysis and insights for therapeutic intervention

Our comparative analysis underscores both unique and shared host response pathways across IAV, MPV, and PIV3 infections. While core antiviral mechanisms, such as interferon signaling and viral genome replication inhibition, are universally activated, variations in specific GO terms and associated genes highlight the unique interactions between the host and each virus. This understanding not only deepens our insights into the molecular underpinnings of host-virus interactions but also illuminates potential targets for broad-spectrum and virus-specific therapeutic interventions aimed at enhancing host defenses and mitigating the adverse effects of viral infections.

In conclusion, this extensive GO analysis across early and later stages post-infection offers a valuable framework for future research into the mechanisms of host resistance and virus pathogenesis. It highlights the complexity of the host’s defense strategies, the adaptability of viral mechanisms to circumvent these defenses, and the potential for identifying novel therapeutic targets. These findings contribute significantly to our understanding of viral infections in OTEs, providing a solid foundation for the development of effective antiviral strategies and enhancing our capacity to combat respiratory viral pathogens.

### 4.2 Classifying respiratory virus infections in OTEs using GLMQL-RMAS

Our study showcases the advanced methodology of biomarker identification through differential expression analysis, underscored by the application of Generalized Linear Models with Quasi-Likelihood *F*-tests (GLMQL-RMAS). This rigorous approach allowed us to identify genes with significant differential expression due to viral infection, utilizing both raw *p*-values and adjusted significance levels for an accurate representation of biological differences.

The differential expression analysis, anchored in the GLMQL framework, pinpoints genes with notable differences in expression across experimental conditions. By emphasizing significant p-values and log fold changes (logFC), we unearth potential biomarkers indicative of specific viral infections. The RMAS algorithm refines gene prioritization by integrating the magnitude of expression changes with statistical significance, surpassing traditional methods that rely solely on *p*-values or logFC. The RMAS and MAS calculations, with hyperparameters M and A set to 1, emphasize the equal importance of expression magnitude and statistical robustness, promoting a balanced evaluation of each gene’s potential as a biomarker.

The selection of the top three genes through RMAS ranking, as illustrated in Figures 6, 7, demonstrates a remarkable 92% mean accuracy in distinguishing between eight different groups using a stratified k-fold cross-validation approach. This precision starkly contrasts with the lesser efficiency of prioritizing genes based solely on p-value or logFC, which achieved a mean accuracy of 85% each, as depicted in Figures 8, 9. This comparison underscores the superiority of integrating both *p*-value and logFC information in biomarker identification.

A pivotal aspect of our discussion focuses on the requirement for an adequate sample size to ensure the reliability of biomarkers in prediction models. Adhering to the principle that at least 10 samples per variable (gene) are necessary, our approach is geared towards maximizing the predictive power and generalizability of the model. The utilization of multinomial logistic regression further enhances the model’s robustness, enabling it to generalize across different biological contexts and experimental conditions.

The employment of multinomial logistic regression is crucial in translating differential expression analysis into actionable insights. This statistical model accommodates the complexities of biological data, ensuring that identified biomarkers are not only statistically significant but also biologically relevant and capable of predicting specific viral infections accurately.

Our comprehensive approach, from differential expression analysis through to biomarker identification using MAS and RMAS, followed by validation via multinomial logistic regression, sets a new standard in the field. It highlights the importance of integrating statistical rigor with biological relevance, paving the way for the identification of robust biomarkers that can inform on viral infections with high precision. This methodology not only contributes to our understanding of viral pathogenesis but also opens avenues for the development of targeted diagnostic and therapeutic strategies, underscoring the potential of advanced statistical models in biomedical research.

## 5 Conclusion

Our study provides a comprehensive examination of gene expression dynamics in response to viral infections, focusing specifically on Influenza A virus (IAV), Human metapneumovirus (MPV), and Parainfluenza virus type 3 (PIV3). Utilizing Generalized Linear Models (GLMs) with Quasi-Likelihood (QL) F-tests (GLMQL) to analyze RNA-Seq data from 19,671 genes, our aim was to identify genes differentially expressed under various infection conditions. The application of GLMQL, along with the innovative Magnitude-Altitude Score (MAS) and Relaxed Magnitude-Altitude Score (RMAS) algorithms, facilitated the precise identification of potential biomarkers indicative of specific viral infections. Our Gene Ontology (GO) analysis further enriched our understanding of the host’s defense mechanisms, identifying key biological processes activated in response to these viral infections. This analysis consistently highlighted the activation of interferon-stimulated genes across all three viruses, underlining the host’s reliance on innate immune mechanisms to combat viral threats. Moreover, the analysis of downregulated genes unveiled viral strategies aimed at manipulating host cellular functions, emphasizing the need for targeted therapeutic interventions.

The GO analysis offered deep insights into the host’s comprehensive response to viral infection, pinpointing critical biological processes, cellular components, and molecular functions impacted by IAV, MPV, and PIV3. Key findings included the activation of a broad range of interferon-stimulated genes (e.g., IFIT1, IFIT2, IFIT3, OAS1) and innate immune response genes (e.g., CGAS, DDX60), showcasing the host’s robust defense mechanisms against viral entry, replication, and spread. Notably, the analysis also revealed significant changes in cellular functions and structures, such as cilium assembly and mitochondrial ribosome assembly, indicating a strategic shift in cellular priorities to support viral replication. The stability of the MAS/RMAS ranking method, even under stringent statistical corrections, underscores the reliability of our approach in identifying key biomarkers.

Our implementation of the GLMQL-RMAS methodology enabled the precise identification of genes with significant differential expression, setting the stage for biomarker identification. This rigorous statistical framework, combined with the strategic use of the MAS and RMAS algorithms, allowed for the nuanced prioritization of genes based on both the magnitude of expression changes and their statistical significance. The classification of respiratory virus infections in OTEs, leveraging a multinomial logistic regression model validated through stratified k-fold cross-validation, achieved remarkable accuracy. This method not only demonstrated a 92% mean accuracy in distinguishing between different viral infections but also underscored the superior efficacy of integrating both *p*-value and logFC information over traditional methods. Crucially, our study highlights the importance of having an adequate sample size for each variable (gene) used as a predictor in the model, ensuring the reliability and generalizability of our findings.

## 6 Limitations of study

While this study offers valuable insights into the dynamics of gene expression in human lung organ tissue equivalents (OTEs) infected with respiratory viruses, it is important to acknowledge several limitations that should be considered when interpreting the findings:

### 6.1 Model specificity

The use of OTEs as a model system, while advantageous for controlled experiments, does not fully recapitulate the complexity of the human lung. Therefore, the observed gene expression patterns may not fully represent the *in vivo* response.

### 6.2 Limited virus selection

This study focuses on a specific set of respiratory viruses (Influenza A, Human metapneumovirus, and Parainfluenza virus type 3). As a result, the findings may not be generalizable to other respiratory pathogens with distinct mechanisms of infection and gene expression modulation.

### 6.3 Short-term observation

The examination of gene expression changes is restricted to the first 72 h post-infection. Longer-term effects, potential recovery processes, and late-stage alterations in gene expression are not captured.

### 6.4 RNA-Seq constraints

RNA-Seq, while a powerful tool, has inherent limitations, including biases in sequence representation and challenges in detecting lowly expressed genes or splice variants.

### 6.5 Clinical translation

Extrapolating findings from the OTE model to human disease requires caution. Factors such as host genetics, comorbidities, and environmental influences, which play a crucial role in clinical outcomes, are not considered in this model.

Despite these limitations, this study provides valuable insights into the early gene expression responses to respiratory viruses within an OTE model. It serves as a foundation for further investigations in the field of viral pathogenesis and host-virus interactions.

## Data availability statement

The datasets presented in this article are not readily available because the current study is part of the Pathogenesis and Toxicity Forecasting Using Multi-Organoid Systems (PATMOS), funded by the US government. In recognition of the significance of data sharing and transparency in advancing scientific research, we understand the interest in accessing these datasets. However, due to specific governance and the sensitive nature of the data under the PATMOS project, these datasets are not publicly available. Researchers who satisfy the established criteria for data access can request the data. These criteria are designed to ensure that data sharing is conducted responsibly and in a manner that respects the guidelines. Requests to access the datasets should be directed to Mostafa Rezapour (mrezapou@wakehealth.edu). Each request will be reviewed promptly, and access will be granted where it is deemed reasonable and permissible under our agreements. We assure prospective researchers that all requests will be considered carefully, with the aim of fostering scientific collaboration and adhering to the principles of transparency and data sharing.

## Author contributions

MR: Conceptualization, Formal analysis, Investigation, Methodology, Software, Validation, Visualization, Writing – original draft, Writing – review & editing. SW: Conceptualization, Data curation, Investigation, Methodology, Supervision, Validation, Writing – original draft, Writing – review & editing. DO: Conceptualization, Data curation, Investigation, Methodology, Supervision, Validation, Writing – original draft, Writing – review & editing. PM: Conceptualization, Funding acquisition, Investigation, Methodology, Project administration, Resources, Supervision, Validation, Writing – review & editing. AA: Conceptualization, Funding acquisition, Investigation, Methodology, Project administration, Resources, Supervision, Validation, Writing – review & editing. MG: Conceptualization, Formal analysis, Investigation, Methodology, Project administration, Resources, Supervision, Validation, Writing – original draft, Writing – review & editing.
