# Supplementary material for: Analysis of gene expression dynamics and differential expression in viral infections using generalized linear models and quasi-likelihood methods
Source: Front Microbiol. 2024 Apr 9;15:1342328. doi: 10.3389/fmicb.2024.1342328 (PMC11037428; doi:10.3389/fmicb.2024.1342328)
Supplement: Supplementary file 1 [file Table_1.DOCX]

**Supplementary Material (SM) for**

Analysis of Gene Expression Dynamics and Differential Expression in Viral Infections Using Generalized Linear Models and Quasi-Likelihood Methods

Mostafa Rezapour, PhD^1*^; Stephen J. Walker, PhD ^2^; David A. Ornelles, PhD ^3^; Patrick M. McNutt, PhD ^2^; Anthony Atala, MD ^2^; Metin Nafi Gurcan, PhD ^1^

^1^Center for Artificial Intelligence Research, Wake Forest University School of Medicine, Winston-Salem, NC, USA

^2^ Wake Forest Institute for Regenerative Medicine, Wake Forest University School of Medicine, Winston-Salem, NC, USA

^3^ Microbiology Immunology, Wake Forest University School of Medicine, Winston-Salem, NC, USA

*** Correspondence:**Mostafa Rezapour, PhD
[mrezapou@wakehealth.edu](mailto:mrezapou@wakehealth.edu)

**Table S1. Top 10 Significant P-Values and Q-Values for GO Biological Process 2023 in Upregulated Significant Genes When Comparing IAV-None-24 to Mock-24.**

| **Term** | **p-value** | **q-value** | **Overlap genes** |
| --- | --- | --- | --- |
| Defense Response To Virus (GO:0051607) | 1.744462e-44 | 5.899769e-41 | IFITM3, RTP4, IFITM1, IFITM2, IFIT5, CGAS, DDX60L, IFIT1, TNF, IFI44L, IFIT3, IFIT2, OASL, IFIH1, TRIM5, DHX58, CASP1, TRIM25, TRIM26, TRIM21, IKBKE, GBP5, GBP7, RSAD2, RIPK3, NT5C3A, PLSCR1, AIM2, IFI27, OAS1, OAS2, IRF1, OAS3, IFIT1B, IRF2, IRF7, TLR3, TLR2, IFI6, DDX60, SAMHD1, USP18, IFNL2, IFNL1, IFI16, PMAIP1, GBP2, IFNL3, APOBEC3F, APOBEC3G, MLKL, IFNB1, STAT1, MX2, MX1, EIF2AK2, IFNLR1, ISG15, PML, BST2, ISG20, CXCL10, ZNFX1, TRIM31, SHFL, APOBEC3A, MYD88, APOBEC3B |
| Defense Response To Symbiont (GO:0140546) | 6.415411e-43 | 1.084846e-39 | IFITM3, RTP4, IFITM1, IFITM2, IFIT5, CGAS, DDX60L, IFIT1, IFI44L, IFIT3, IFIT2, OASL, IFIH1, TRIM5, CASP1, IKBKE, GBP5, GBP7, RSAD2, RIPK3, NT5C3A, PLSCR1, AIM2, IFI27, OAS1, OAS2, IRF1, OAS3, IFIT1B, IRF2, IRF7, TLR3, TLR2, IFI6, DDX60, SAMHD1, IFNL2, IFNL1, IFI16, PMAIP1, GBP2, IFNL3, APOBEC3F, APOBEC3G, MLKL, IFNB1, STAT1, MX2, MX1, EIF2AK2, IFNLR1, ISG15, BST2, ISG20, ZNFX1, TRIM31, SHFL, APOBEC3A, MYD88, APOBEC3B |
| Negative Regulation Of Viral Process (GO:0048525) | 9.861638e-34 | 1.111735e-30 | IFITM3, IFITM1, IFITM2, IFIT5, ZC3HAV1, IFIT1, TNF, OASL, IFIH1, ZFP36, IFI16, CCL5, TRIM21, IFNL3, N4BP1, APOBEC3F, RSAD2, APOBEC3G, IFNB1, STAT1, MX1, EIF2AK2, ISG15, BST2, ISG20, FAM111A, PLSCR1, ZNFX1, OAS1, TNIP1, OAS2, OAS3, TRIM14, TRIM31, SHFL, APOBEC3A |
| Negative Regulation Of Viral Genome Replication (GO:0045071) | 1.429776e-30 | 1.208876e-27 | IFITM3, IFITM1, IFITM2, IFIT5, ZC3HAV1, IFIT1, TNF, OASL, IFIH1, IFI16, CCL5, IFNL3, N4BP1, APOBEC3F, RSAD2, APOBEC3G, IFNB1, MX1, EIF2AK2, ISG15, BST2, ISG20, FAM111A, PLSCR1, ZNFX1, OAS1, TNIP1, OAS2, OAS3, SHFL, APOBEC3A, APOBEC3B |
| Regulation Of Viral Genome Replication (GO:0045069) | 6.718836e-29 | 4.544621e-26 | IFITM3, IFITM1, CXCL8, IFITM2, STAU1, IFIT5, ZC3HAV1, IFIT1, TNF, OASL, IFIH1, IFI16, CCL5, IFNL3, N4BP1, GBP7, APOBEC3F, RSAD2, APOBEC3G, IFNB1, MX1, EIF2AK2, ISG15, BST2, ISG20, FAM111A, PLSCR1, ZNFX1, OAS1, TNIP1, OAS2, OAS3, SHFL, APOBEC3A |
| Response To Cytokine (GO:0034097) | 2.492655e-20 | 1.405027e-17 | IFITM3, CD274, CSF3, IFITM1, SP100, IFITM2, CALCOCO2, ADAR, CXCL16, RELB, NUB1, IRAK2, LAMP3, KYNU, RIPK1, LGALS9, JAK2, TRIM21, IKBKE, MCL1, GCH1, STAT1, MX2, MX1, EIF2AK2, LIFR, IFNLR1, ISG15, PML, BST2, CH25H, PLSCR1, IL1B, XAF1, SHFL, MYD88 |
| Response To Type II Interferon (GO:0034341) | 9.547845e-20 | 4.612973e-17 | IFITM3, IFITM1, SP100, IFITM2, CALCOCO2, CX3CL1, CXCL16, NUB1, KYNU, CCL5, CCL4, CASP1, CCL3, LGALS9, GBP2, TRIM21, GBP1, GBP4, HLA-DPA1, GBP6, GBP5, CCL22, GCH1, CCL20, STAT1, BST2, CD47, SHFL, TLR2 |
| Positive Regulation Of Intracellular Signal Transduction (GO:1902533) | 1.923014e-18 | 8.129543e-16 | RET, CSF3, BMPR2, PLEKHF1, NCF1, RNF13, TRAF3IP2, RTKN2, HTR2B, IFIT5, SECTM1, IFI35, TNF, CX3CL1, BBC3, CASP10, TRIM5, NUP62, TNFSF10, CASP1, TRIM25, LGALS9, SOX9, JAK2, IKBKE, TRIM21, TRIM22, NKX3-1, RIPK2, ARRDC3, IRAK4, TICAM1, IL23A, IL1B, PELI1, ADAM8, RBCK1, NUPR1, TMEM106A, S100A8, TLR3, HBEGF, BIRC3, TLR2, NOD2, MST1R, CLEC7A, CCL5, CCL4, MIER1, CCL3, PMAIP1, RICTOR, RIPK1, APOL2, LYN, TBX1, TCF7L2, NDFIP1, DAB2IP, LIF, EIF2AK2, SEPTIN4, CFLAR, BST2, BMP2, TNIP2, HPSE, TRIM38, PIK3AP1, MYD88, TRIM34 |
| Regulation Of I-kappaB kinase/NF-kappaB Signaling (GO:0043122) | 1.211121e-17 | 4.551124e-15 | TRAF3IP2, IFIT5, HTR2B, SECTM1, TNFAIP3, NOD2, TNF, CX3CL1, CASP10, TRIM5, CLEC7A, ZC3H12A, MIER1, NUP62, TNFSF10, CASP1, TRIM25, RIPK1, LGALS9, TRIM21, IKBKE, TRIM22, APOL2, GBP7, NDFIP1, TLE1, RIPK2, STAT1, DAB2IP, NR1D1, IRAK4, CFLAR, TICAM1, BST2, TNIP1, TNIP2, IL1B, PELI1, RBCK1, TRIM38, OPTN, TMEM106A, MYD88, BIRC3, TRIM34 |
| Cellular Response To Cytokine Stimulus (GO:0071345) | 4.127660e-16 | 1.395974e-13 | RIPOR2, IFNA7, CSF3, IFITM2, CXCL8, IFNA1, IFIT1, CX3CL1, ZFP36, ZC3H12A, CASP1, LGALS9, SOX9, JAK2, HLA-DPA1, NKX3-1, GBP6, GBP5, LIFR, IRAK4, EREG, IL1A, AIM2, OAS1, IL1B, IRF1, CD47, TLR2, IL2RG, ZFP36L2, SOCS1, IFI16, CCL5, CCL4, CCL3, RIPK1, GBP2, GBP1, DUOX2, GBP4, GBP3, CCL22, PNPT1, CCL20, IFNB1, STAT1, DAB2IP, IL36G, NR1D1, NFKBIA |

**Table S2. Top 10 Significant P-Values and Q-Values for GO Biological Process 2023 in Downregulated Significant Genes When Comparing IAV-None-24 to Mock-24.**

| **Term** | **p-value** | **q-value** | **Overlap genes** |
| --- | --- | --- | --- |
| Cilium Assembly (GO:0060271) | 3.878250e-10 | 0.000001 | UNC119B, GALNT11, LRGUK, TRAF3IP1, ARL3, DNAH5, FNBP1L, SPATA6, KIF3B, TCTN3, TEKT1, CFAP43, CFAP20, DYNC2H1, BBS2, RSPH4A, GBF1, RFX2, CSNK1D, IFT80, RPGRIP1L, TMEM231, EHD2, AHI1, IFT88, UBXN10, TMEM216, TTC30A, CEP83, WDPCP, CCDC65, B9D2, CEP41, BBOF1, ATP6V0D1, IFT46, GAS8, SPACA9 |
| Plasma Membrane Bounded Cell Projection Assembly (GO:0120031) | 4.085312e-09 | 0.000006 | UNC119B, GALNT11, SPEF1, TRAF3IP1, ARL3, DNAH5, FNBP1L, SLC9A3R1, KIF3B, TCTN3, TEKT1, ARFIP2, INPP5K, SRGAP3, CFAP43, CFAP20, DYNC2H1, BBS2, SPAG6, GBF1, RFX2, IFT80, EMP2, PARVB, TMEM231, EHD2, AHI1, IFT88, UBXN10, TMEM216, TTC30A, CEP83, WDPCP, CCDC65, B9D2, CEP41, ATP6V0D1, ARHGEF7, IFT46 |
| Cilium Organization (GO:0044782) | 1.120370e-08 | 0.000011 | UNC119B, GALNT11, TRAF3IP1, ARL3, DNAH5, FNBP1L, TTC29, SLC9A3R1, KIF3B, TCTN3, TEKT1, CFAP43, CFAP20, DYNC2H1, BBS2, GBF1, RFX2, IFT80, TMEM231, ARMC2, EHD2, AHI1, IFT88, UBXN10, TMEM216, TTC30A, CEP83, WDPCP, CCDC65, B9D2, CEP41, MNS1, ATP6V0D1, IFT46 |
| Organelle Assembly (GO:0070925) | 1.101206e-07 | 0.000084 | UNC119B, GALNT11, TRAF3IP1, ARL3, DNAH5, FNBP1L, FXR1, AP4M1, KIF3B, TCTN3, CHMP1B, RB1CC1, TP53INP2, TEKT1, TP53INP1, CFAP43, CFAP20, DYNC2H1, BBS2, YTHDF2, GBF1, RFX2, IFT80, TMEM231, EHD2, AHI1, IFT88, ATG16L1, UBXN10, TMEM216, TTC30A, CEP83, WDPCP, CCDC65, B9D2, CEP41, ATP6V0D1, IFT46, VPS25, ATG2B |
| Mitochondrial Ribosome Assembly (GO:0061668) | 1.135464e-06 | 0.000694 | DHX30, MTERF3, FASTKD2, NOA1, MRPS2, MRM2 |
| Cilium Movement (GO:0003341) | 5.351788e-06 | 0.002609 | DNAI4, RSPH4A, SPEF1, DNAH7, DNAAF2, SPAG6, DNAH5, DNAAF5, TTC29, CABYR, TEKT1, HYDIN, GAS8 |
| Regulation Of Protein Localization To Cilium (GO:1903564) | 5.972299e-06 | 0.002609 | GSK3B, CROCC, ENTR1, GAS8, LZTFL1 |
| Positive Regulation Of Protein Localization To Cilium (GO:1903566) | 2.983244e-05 | 0.010213 | GSK3B, CROCC, ENTR1, GAS8 |
| Organelle Organization (GO:0006996) | 3.148164e-05 | 0.010213 | DYRK3, STOML2, PEX11A, FNBP1L, TTC29, PHB2, SYNE1, SLC9A3R1, FAM174B, CHMP1B, TMED3, TRAPPC12, PACSIN2, MLST8, CLASP2, DYNC2H1, COG8, JAGN1, YTHDF2, AGTPBP1, VPS13D, GBF1, COG2, LIG4, CSNK1D, PEX2, MTOR, ARMC2, SEC23IP, ABI2, VAPA, NOA1, KAT6A, RAB34, GOLGB1, TRIP11, MTFR1, WDPCP, MNS1, ARHGEF7, BRWD1 |
| Cilium-Dependent Cell Motility (GO:0060285) | 3.339782e-05 | 0.010213 | DNAH3, DNAH2, DNAH7, DNAAF2, TEKT1, CCDC65, GAS8 |

**Table S3. Top 10 Significant P-Values and Q-Values for GO Biological Process 2023 in Upregulated Significant Genes When Comparing MPV-None-24 to Mock-24.**

| **Term** | **p-value** | **q-value** | **Overlap genes** |
| --- | --- | --- | --- |
| Defense Response To Symbiont (GO:0140546) | 7.474605e-14 | 5.060307e-11 | IFITM1, RSAD2, MX2, MX1, ISG15, IFIT1, TANK, IFIT3, IFIT2, PLSCR1, OAS1, OAS2, OAS3 |
| Negative Regulation Of Viral Genome Replication (GO:0045071) | 1.381954e-12 | 3.966404e-10 | IFITM1, PLSCR1, RSAD2, OAS1, OAS2, OAS3, MX1, ISG15, IFIT1 |
| Defense Response To Virus (GO:0051607) | 1.757639e-12 | 3.966404e-10 | IFITM1, RSAD2, MX2, MX1, ISG15, IFIT1, TANK, IFIT3, IFIT2, PLSCR1, OAS1, OAS2, OAS3 |
| Negative Regulation Of Viral Process (GO:0048525) | 5.257900e-12 | 8.898996e-10 | IFITM1, PLSCR1, RSAD2, OAS1, OAS2, OAS3, MX1, ISG15, IFIT1 |
| Regulation Of Viral Genome Replication (GO:0045069) | 1.269322e-11 | 1.718661e-09 | IFITM1, PLSCR1, RSAD2, OAS1, OAS2, OAS3, MX1, ISG15, IFIT1 |
| Positive Regulation Of Type I Interferon Production (GO:0032481) | 3.483968e-07 | 3.931077e-05 | OAS1, OAS2, OAS3, ISG15, PTPN11, TANK |
| Positive Regulation Of Interferon-Beta Production (GO:0032728) | 4.700003e-07 | 4.545574e-05 | OAS1, OAS2, OAS3, ISG15, PTPN11 |
| Interleukin-27-Mediated Signaling Pathway (GO:0070106) | 7.900057e-07 | 5.942599e-05 | OAS1, OAS2, MX1 |
| Regulation Of Ribonuclease Activity (GO:0060700) | 7.900057e-07 | 5.942599e-05 | OAS1, OAS2, OAS3 |
| Regulation Of Nuclease Activity (GO:0032069) | 1.575049e-06 | 1.016462e-04 | OAS1, OAS2, OAS3 |

**Table S4. Top 10 Significant P-Values and Q-Values for GO Biological Process 2023 in Downregulated Significant Genes When Comparing MPV-None-24 to Mock-24.**

| **Term** | **p-value** | **q-value** | **Overlap genes** |
| --- | --- | --- | --- |
| Lysosomal Lumen Acidification (GO:0007042) | 0.000003 | 0.003232 | ATP6V0B, RNASEK, CCDC115, ATP6V1F |
| Ribosome Assembly (GO:0042255) | 0.000011 | 0.004007 | EFNA1, RPS28, EIF6, MRPS2, RPS27L |
| Regulation Of Lysosomal Lumen pH (GO:0035751) | 0.000013 | 0.004007 | ATP6V0B, RNASEK, CCDC115, ATP6V1F |
| Golgi Lumen Acidification (GO:0061795) | 0.000016 | 0.004007 | ATP6V0B, RNASEK, ATP6V1F |
| Vacuolar Acidification (GO:0007035) | 0.000024 | 0.004772 | ATP6V0B, RNASEK, CCDC115, ATP6V1F |
| Endosomal Lumen Acidification (GO:0048388) | 0.000032 | 0.005202 | ATP6V0B, RNASEK, ATP6V1F |
| Response To Cadmium Ion (GO:0046686) | 0.000246 | 0.030167 | NCF1, MT1F, MT1G |
| Endosome Organization (GO:0007032) | 0.000270 | 0.030167 | VPS18, ATP6V0B, RNASEK, ATP6V1F |
| Cellular Response To Cadmium Ion (GO:0071276) | 0.000284 | 0.030167 | NCF1, MT1F, MT1G |
| Regulation Of Neutrophil Extravasation (GO:2000389) | 0.000341 | 0.030167 | MDK, CD99 |

**Table S5. Top 10 Significant P-Values and Q-Values for GO Biological Process 2023 in Upregulated Significant Genes When Comparing PIV3-None-24 to Mock-24.**

| **Term** | **p-value** | **q-value** | **Overlap genes** |
| --- | --- | --- | --- |
| Defense Response To Virus (GO:0051607) | 3.544457e-33 | 4.462471e-30 | RTP4, IFITM1, IFIT5, DDX60L, IFIT1, DDX60, TANK, USP18, IFI44L, IFIT3, IFIT2, OASL, IFIH1, IFNL2, IFNL1, PMAIP1, TRIM25, GBP5, RSAD2, STAT1, MX2, MX1, EIF2AK2, ISG15, NT5C3A, CXCL10, PLSCR1, ZNFX1, OAS1, OAS2, OAS3, IRF2, F2RL1 |
| Defense Response To Symbiont (GO:0140546) | 2.897708e-32 | 1.824107e-29 | RTP4, IFITM1, IFIT5, DDX60L, IFIT1, DDX60, TANK, IFI44L, IFIT3, IFIT2, OASL, IFIH1, IFNL2, IFNL1, PMAIP1, GBP5, RSAD2, STAT1, MX2, MX1, EIF2AK2, ISG15, NT5C3A, PLSCR1, ZNFX1, OAS1, OAS2, OAS3, IRF2, F2RL1 |
| Negative Regulation Of Viral Genome Replication (GO:0045071) | 1.044136e-20 | 4.381890e-18 | IFITM1, RSAD2, MX1, IFIT5, EIF2AK2, ISG15, ZC3HAV1, IFIT1, OASL, IFIH1, PLSCR1, ZNFX1, OAS1, OAS2, OAS3, TASOR |
| Negative Regulation Of Viral Process (GO:0048525) | 1.340752e-19 | 4.220016e-17 | IFITM1, RSAD2, STAT1, MX1, IFIT5, EIF2AK2, ISG15, ZC3HAV1, IFIT1, OASL, IFIH1, PLSCR1, ZNFX1, OAS1, OAS2, OAS3 |
| Regulation Of Viral Genome Replication (GO:0045069) | 7.091943e-19 | 1.785751e-16 | IFITM1, CXCL8, RSAD2, MX1, IFIT5, EIF2AK2, ISG15, ZC3HAV1, IFIT1, OASL, IFIH1, PLSCR1, ZNFX1, OAS1, OAS2, OAS3 |
| Antiviral Innate Immune Response (GO:0140374) | 8.350296e-12 | 1.752171e-09 | IFIH1, CXCL10, OAS1, MX1, TRIM25, EIF2AK2, IFIT1, USP18, IFIT3, IFIT2 |
| Interleukin-27-Mediated Signaling Pathway (GO:0070106) | 5.584486e-11 | 1.004410e-08 | OAS1, OAS2, STAT1, MX1, OASL |
| Cytokine-Mediated Signaling Pathway (GO:0019221) | 1.227468e-08 | 1.931728e-06 | LYN, IL11, CXCL6, CXCL8, STAT1, MX1, PTPN11, CXCL5, OASL, CXCL10, CXCL11, OAS1, OAS2, TRAF5, IL13RA2 |
| Regulation Of Ribonuclease Activity (GO:0060700) | 3.150419e-08 | 4.407087e-06 | OAS1, OAS2, OAS3, OASL |
| Regulation Of Nuclease Activity (GO:0032069) | 9.384887e-08 | 1.181557e-05 | OAS1, OAS2, OAS3, OASL |

**Table S6. Top 10 Significant P-Values and Q-Values for GO Biological Process 2023 in Downregulated Significant Genes When Comparing PIV3-None-24 to Mock-24.**

| **Term** | **p-value** | **q-value** | **Overlap genes** |
| --- | --- | --- | --- |
| Carboxylic Acid Catabolic Process (GO:0046395) | 0.000039 | 0.027633 | BCKDK, TST, ETFB |
| Negative Regulation Of Cellular Response To Hypoxia (GO:1900038) | 0.000153 | 0.046339 | ENO1, NOL3 |
| Glycolytic Process (GO:0006096) | 0.000201 | 0.046339 | LDHA, PGAM4, ENO1 |
| Response To Hydroperoxide (GO:0033194) | 0.000319 | 0.046339 | GPX3, PRKD1 |
| Cellular Response To Vascular Endothelial Growth Factor Stimulus (GO:0035924) | 0.000325 | 0.046339 | MT1G, PRKD1, PGF |
| Amino Acid Catabolic Process (GO:0009063) | 0.000453 | 0.053824 | BCKDK, HAL, ETFB |
| Carbohydrate Catabolic Process (GO:0016052) | 0.000567 | 0.057803 | LDHA, PGAM4, ENO1 |
| Positive Regulation Of B Cell Differentiation (GO:0045579) | 0.000679 | 0.059032 | XBP1, PPP2R3C |
| Positive Regulation Of Vasculature Development (GO:1904018) | 0.000769 | 0.059032 | XBP1, GRN, PRKD1, PGF |
| Pentose-Phosphate Shunt (GO:0006098) | 0.000828 | 0.059032 | TALDO1, PGLS |

**Table S7. Top 10 Significant P-Values and Q-Values for GO Biological Process 2023 in Upregulated Significant Genes When Comparing IAV-None-72 to Mock-72.**

| **Term** | **p-value** | **q-value** | **Overlap genes** |
| --- | --- | --- | --- |
| Defense Response To Virus (GO:0051607) | 4.106471e-37 | 1.530482e-33 | IFITM3, RTP4, IFITM1, CD40, IFITM2, IFIT5, DDX60L, IFIT1, TNF, IFI44L, IFIT3, IFIT2, OASL, IFIH1, TRIM5, DHX58, CASP1, TRIM25, TRIM21, NCK1, GBP5, GBP7, RSAD2, RNASE1, NT5C3A, PLSCR1, AIM2, IFI27, OAS1, OAS2, IRF1, OAS3, IFIT1B, IRF7, TLR3, TLR2, IFI6, DDX60, SAMHD1, TANK, USP18, IFNL2, IFNL1, IFI16, PMAIP1, IFNL3, APOBEC3F, APOBEC3G, MLKL, IFNB1, STAT1, MX2, STAT2, MX1, EIF2AK2, ISG15, PML, BST2, ISG20, CXCL10, ZNFX1, SHFL, APOBEC3A, MYD88, APOBEC3B |
| Defense Response To Symbiont (GO:0140546) | 8.983444e-36 | 1.674065e-32 | IFITM3, RTP4, IFITM1, CD40, IFITM2, IFIT5, DDX60L, IFIT1, IFI44L, IFIT3, IFIT2, OASL, IFIH1, TRIM5, CASP1, GBP5, GBP7, RSAD2, RNASE1, NT5C3A, PLSCR1, AIM2, IFI27, OAS1, OAS2, IRF1, OAS3, IFIT1B, IRF7, TLR3, TLR2, IFI6, DDX60, SAMHD1, TANK, IFNL2, IFNL1, IFI16, PMAIP1, IFNL3, APOBEC3F, APOBEC3G, MLKL, IFNB1, STAT1, MX2, STAT2, MX1, EIF2AK2, ISG15, BST2, ISG20, ZNFX1, SHFL, APOBEC3A, MYD88, APOBEC3B |
| Negative Regulation Of Viral Process (GO:0048525) | 1.457869e-28 | 1.555334e-25 | IFITM3, IFITM1, IFITM2, IFIT5, ZC3HAV1, IFIT1, TNF, OASL, IFIH1, ZFP36, IFI16, CCL5, TRIM21, IFNL3, N4BP1, APOBEC3F, RSAD2, APOBEC3G, IFNB1, STAT1, MX1, EIF2AK2, ISG15, BST2, ISG20, PLSCR1, ZNFX1, OAS1, TNIP1, OAS2, OAS3, TRIM14, SHFL, APOBEC3A |
| Negative Regulation Of Viral Genome Replication (GO:0045071) | 1.669261e-28 | 1.555334e-25 | IFITM3, IFITM1, IFITM2, IFIT5, ZC3HAV1, IFIT1, TNF, OASL, RESF1, IFIH1, IFI16, CCL5, IFNL3, N4BP1, APOBEC3F, RSAD2, APOBEC3G, IFNB1, MX1, EIF2AK2, ISG15, BST2, ISG20, PLSCR1, ZNFX1, OAS1, TNIP1, OAS2, OAS3, SHFL, APOBEC3A, APOBEC3B |
| Regulation Of Viral Genome Replication (GO:0045069) | 3.590358e-24 | 2.676253e-21 | IFITM3, IFITM1, CXCL8, IFITM2, IFIT5, ZC3HAV1, IFIT1, TNF, OASL, IFIH1, IFI16, CCL5, IFNL3, N4BP1, GBP7, APOBEC3F, RSAD2, APOBEC3G, IFNB1, MX1, EIF2AK2, ISG15, BST2, ISG20, PLSCR1, ZNFX1, OAS1, TNIP1, OAS2, OAS3, SHFL, APOBEC3A |
| Response To Cytokine (GO:0034097) | 3.350446e-18 | 2.081186e-15 | IFITM3, CD274, IFITM1, CD40, SP100, IFITM2, CALCOCO2, ADAR, CXCL16, RELB, NUB1, IRAK2, LAMP3, UBD, SMPD1, TIMP2, LGALS9, JAK2, TRIM21, MCL1, GCH1, STAT1, SPHK1, MX2, MX1, EIF2AK2, LIFR, ISG15, OSMR, PML, BST2, PLSCR1, COL3A1, XAF1, SHFL, MYD88 |
| Regulation Of Inflammatory Response (GO:0050727) | 1.098193e-16 | 5.847091e-14 | PTGER4, SEMA7A, TNFAIP6, SERPINE1, CXCL17, TNFAIP3, IFI35, NOD2, METRNL, ETS1, TNF, USP18, CX3CL1, CAMK2N1, MDK, TMSB4X, CCL5, CASP1, CCL3, CCN4, CCN3, SLC39A8, JAK2, SNX6, IL15, SPHK1, WNT5A, MMP3, NR1D1, KLF4, IL22RA1, NFKBIA, ACE2, CYLD, PSMA6, FNDC4, AIM2, SELENOS, TNIP1, NINJ1, CD47, TEK, PIK3AP1, TLR3, MYD88, BIRC2, TLR2, BIRC3 |
| Cellular Response To Lipopolysaccharide (GO:0071222) | 1.123715e-15 | 5.235105e-13 | CD274, CXCL9, CXCL8, SERPINE1, TNFAIP3, NOD2, TNF, ZFP36, CASP7, CCL5, ZC3H12A, ANKRD1, CASP1, CCL3, CCL2, IL36RN, GBP3, LYN, WNT5A, DAB2IP, IL36G, NR1D1, PDCD1LG2, TICAM1, CXCL10, CXCL11, SELENOS, TNIP1, AXL, TNIP2, CD68, MYD88, CHMP5 |
| Response To Lipopolysaccharide (GO:0032496) | 2.337121e-15 | 9.678278e-13 | CD274, CXCL9, CXCL8, SERPINE1, TNFAIP3, NOD2, ZFP36, CASP7, ZC3H12A, ANKRD1, CASP1, CCL2, IL12A, IL36RN, LGALS9, JAK2, GBP3, GCH1, WNT5A, DAB2IP, ERBIN, IL36G, NR1D1, PDCD1LG2, TICAM1, CXCL10, CXCL11, SELENOS, TNIP1, AXL, TNIP2, PELI1, TAB2, TRIB1, CD68, MYD88, CHMP5 |
| Regulation Of I-kappaB kinase/NF-kappaB Signaling (GO:0043122) | 3.316584e-15 | 1.236091e-12 | CD40, IFIT5, HTR2B, SECTM1, TNFAIP3, NOD2, TANK, TNF, CX3CL1, CASP10, TRIM5, UBD, ZC3H12A, MIER1, NUP62, TNFSF10, CASP1, TRIM25, SLC39A8, LGALS9, TRIM21, TRIM22, APOL2, GBP7, NDFIP1, STAT1, WNT5A, DAB2IP, SHISA5, NR1D1, TRAF1, CFLAR, TICAM1, BST2, TNIP1, TNIP2, PELI1, TAB2, TRIM38, OPTN, TMEM9B, MYD88, BIRC2, BIRC3, TRIM34 |

**Table S8. Top 10 Significant P-Values and Q-Values for GO Biological Process 2023 in Downregulated Significant Genes When Comparing IAV-None-72 to Mock-72.**

| **Term** | **p-value** | **q-value** | **Overlap genes** |
| --- | --- | --- | --- |
| Translation (GO:0006412) | 1.716745e-34 | 5.337361e-31 | RPL4, RPL5, RPL3, RPL31, RPLP1, RPLP0, MRPL37, RPL8, APEH, RPL10A, RPL9, RPL6, EEF1B2, RPS4X, RPS14, RPL7A, RPS17, RPS16, RPL18A, NARS1, RPS18, RACK1, RPS10, RARS2, RPS9, MRPS27, RARS1, RPL21, MRPS24, RPS8, PABPC4, RPS5, RPL22, RPS6, MRPS2, RPL13A, RPSA, RPS3A, MRPL45, DARS1, EEF1A1, EEF1G, SARS1, EEF1D, LARS1, EEF1A2, RPL37A, RPL29, UBA52, RPL10, MRPS34, RPL11, RPS4Y1, RPS3, RPL13, IARS2, RPL15, RPS2, RPL18, RPS27A, RPL17, RPL19, EEF2, WARS2, GARS1, EPRS1, RPS21, RPS24, RPS23 |
| Cytoplasmic Translation (GO:0002181) | 8.489110e-34 | 1.319632e-30 | RPL4, RPL5, RPL3, RPL10, RPL31, RPLP1, RPLP0, RPL11, RPL10A, RPL8, RPL9, RPL6, RPS4X, RPL7A, RPS14, RPS17, RPS16, RPL18A, RPS18, RACK1, RPS3, RPL13, RPL15, RPS2, RPL18, RPS27A, RPS10, RPL17, RPL19, RPS9, RPL21, RPS8, RPS5, RPL22, RPS6, RPL13A, RPS3A, RPSA, SARS1, RPL37A, RPL29, UBA52, RPS21, RPS24, RPS23 |
| Macromolecule Biosynthetic Process (GO:0009059) | 3.075262e-27 | 3.186997e-24 | RPL4, RPL5, RPL3, RPL31, RPLP1, RPLP0, MRPL37, RPL10A, RPL8, RPL9, RPL6, EEF1B2, RPS4X, RPS14, RPL7A, RPS17, RPS16, RPL18A, RPS18, RPS10, RPS9, RPL21, RPS8, RPS5, PABPC4, RPL22, RPS6, RPL13A, RPSA, RPS3A, DARS1, EEF1A1, EEF1G, SARS1, EEF1D, EEF1A2, RPL37A, RPL29, RPL10, RPL11, RPS4Y1, RPS3, RPL13, APOE, RPL15, RPS2, RPL18, RPS27A, RPL17, RPL19, EEF2, RPS21, RPS24, RPS23 |
| Peptide Biosynthetic Process (GO:0043043) | 6.476539e-26 | 5.033890e-23 | RPL4, RPL5, RPL3, RPL31, RPLP1, RPLP0, MRPL37, RPL10A, RPL8, RPL9, RPL6, RPS4X, RPS14, RPL7A, RPS17, RPS16, RPL18A, RPS18, RPS10, RPS9, RPL21, RPS8, RPS5, PABPC4, RPL22, RPS6, RPL13A, RPSA, RPS3A, DARS1, EEF1A1, SARS1, EEF1A2, RPL37A, RPL29, RPL10, RPL11, RPS4Y1, RPS3, RPL13, RPL15, RPS2, RPL18, RPS27A, RPL17, RPL19, RPS21, RPS24, RPS23 |
| Cilium Movement (GO:0003341) | 3.222724e-21 | 2.003890e-18 | SPAG17, SPEF2, SPEF1, DNAH7, DNAH5, DNAH9, RSPH9, CFAP206, TTC29, CFAP251, TEKT1, HYDIN, ROPN1L, SPA17, DNAI1, DNAI4, CCDC39, DNAH11, RSPH4A, DNAI3, CFAP73, SPAG6, CFAP91, DNAAF6, NME5, CFAP100, CFAP53, GAS8 |
| Gene Expression (GO:0010467) | 7.381584e-15 | 3.824891e-12 | RPL4, RPL5, RPL3, RPL31, RPLP1, RPLP0, MRPL37, RPL8, RPL10A, RPL9, RPL6, RBM3, RPS4X, RPS14, RPL7A, RPS17, RPS16, RPL18A, RPS18, RPS10, RPS9, RPL21, RPS8, PABPC4, RPS5, RPL22, RPS6, RPL13A, RPSA, RPS3A, DARS1, EEF1A1, SARS1, EEF1A2, RPL37A, RPL29, RPL10, RPL11, RPS4Y1, RPS3, RPL13, RPL15, RPS2, RPL18, RPS27A, RPL17, RPL19, SORL1, RPS21, RPS24, RPS23 |
| Axoneme Assembly (GO:0035082) | 1.386094e-14 | 6.156236e-12 | DNAI4, SPAG17, TTC26, CCDC39, RSPH4A, SPEF1, DNAAF3, CFAP91, DNAAF6, DNAJB13, RSPH9, CFAP206, RP1, RSPH1, HYDIN, CCDC65, HOATZ, GAS8, SPACA9 |
| Cilium Assembly (GO:0060271) | 6.669096e-12 | 2.591777e-09 | UNC119B, SPAG17, CEP126, TTC26, TRAF3IP1, ARL3, DNAH5, RSPH9, CFAP206, SPATA6, HSPB11, CDC14B, DYNC2LI1, ABLIM1, KIF3A, TEKT1, CCDC96, CFAP47, CFAP43, CC2D2A, DYNC2H1, CCDC39, RSPH4A, GSN, DNAAF3, CCDC113, TTC39C, IFT88, RP1, RSPH1, CFAP161, CEP83, IFT27, CCDC65, HOATZ, B9D2, BBOF1, ZMYND10, CIBAR2, GAS8, SPACA9 |
| Axonemal Dynein Complex Assembly (GO:0070286) | 1.157914e-09 | 3.999950e-07 | DNAI4, CCDC39, DNAH2, CFAP73, DNAI3, DNAH7, DNAAF3, DNAH5, DNAAF6, CFAP100, CCDC65, ZMYND10, DNAI1, DNAL1 |
| Cilium Organization (GO:0044782) | 3.851706e-08 | 1.197495e-05 | UNC119B, CEP126, TTC26, TRAF3IP1, ARL3, DNAH5, TTC29, HSPB11, CDC14B, SLC9A3R1, ABLIM1, KIF3A, TEKT1, CCDC96, CFAP47, MARK4, CFAP43, CC2D2A, DYNC2H1, GSN, CCDC113, TTC39C, IQCG, ARMC2, IFT88, CFAP161, CEP83, IFT27, CCDC65, HOATZ, B9D2, MNS1, CIBAR2 |

**Table S9. Top 10 Significant P-Values and Q-Values for GO Biological Process 2023 in Upregulated Significant Genes When Comparing MPV-None-72 to Mock-72.**

| **Term** | **p-value** | **q-value** | **Overlap genes** |
| --- | --- | --- | --- |
| Defense Response To Virus (GO:0051607) | 3.318522e-36 | 6.258732e-33 | IFITM3, RTP4, IFITM1, IFIT5, IFI6, DDX60L, IFIT1, DDX60, SAMHD1, USP18, IFI44L, IFIT3, IFIT2, OASL, IFIH1, IFI16, DHX58, TRIM25, TRIM21, RSAD2, STAT1, MX2, MX1, EIF2AK2, ISG15, PML, BST2, ISG20, NT5C3A, CXCL10, PLSCR1, ZNFX1, IFI27, OAS1, OAS2, IRF1, OAS3, IFIT1B, IRF7, SHFL, APOBEC3A, TLR3 |
| Defense Response To Symbiont (GO:0140546) | 1.218238e-32 | 1.148799e-29 | IFITM3, RTP4, IFITM1, IFIT5, IFI6, DDX60L, IFIT1, DDX60, SAMHD1, IFI44L, IFIT3, IFIT2, OASL, IFIH1, IFI16, RSAD2, STAT1, MX2, MX1, EIF2AK2, ISG15, BST2, ISG20, NT5C3A, PLSCR1, ZNFX1, IFI27, OAS1, OAS2, IRF1, OAS3, IFIT1B, IRF7, SHFL, APOBEC3A, TLR3 |
| Negative Regulation Of Viral Process (GO:0048525) | 3.360369e-26 | 2.112552e-23 | IFITM3, IFITM1, RSAD2, STAT1, MX1, IFIT5, EIF2AK2, ISG15, ZC3HAV1, IFIT1, OASL, IFIH1, BST2, ISG20, PLSCR1, ZNFX1, OAS1, IFI16, OAS2, OAS3, TRIM21, SHFL, APOBEC3A |
| Negative Regulation Of Viral Genome Replication (GO:0045071) | 1.434042e-24 | 6.761510e-22 | IFITM3, IFITM1, RSAD2, MX1, IFIT5, EIF2AK2, ISG15, ZC3HAV1, IFIT1, OASL, IFIH1, BST2, ISG20, PLSCR1, ZNFX1, OAS1, IFI16, OAS2, OAS3, SHFL, APOBEC3A |
| Regulation Of Viral Genome Replication (GO:0045069) | 4.800034e-22 | 1.810573e-19 | IFITM3, IFITM1, RSAD2, MX1, IFIT5, EIF2AK2, ISG15, ZC3HAV1, IFIT1, OASL, IFIH1, BST2, ISG20, PLSCR1, ZNFX1, OAS1, IFI16, OAS2, OAS3, SHFL, APOBEC3A |
| Response To Cytokine (GO:0034097) | 5.825177e-16 | 1.831047e-13 | IFITM3, IFITM1, SP100, IL1R1, STAT1, MX2, MX1, EIF2AK2, ISG15, ADAR, PML, BST2, PLSCR1, COL3A1, LAMP3, SMPD1, TIMP3, LGALS9, XAF1, TRIM21, SHFL |
| Response To Interferon-Beta (GO:0035456) | 1.251634e-11 | 3.372260e-09 | IFITM3, BST2, IFITM1, PLSCR1, IFI16, OAS1, STAT1, IRF1, XAF1, SHFL |
| Antiviral Innate Immune Response (GO:0140374) | 1.015002e-10 | 2.392867e-08 | IFIH1, CXCL10, OAS1, DHX58, MX1, TRIM25, EIF2AK2, IFIT1, USP18, IFIT3, IFIT2 |
| Interleukin-27-Mediated Signaling Pathway (GO:0070106) | 9.390252e-10 | 1.967779e-07 | OAS1, OAS2, STAT1, MX1, OASL |
| Response To Type I Interferon (GO:0034340) | 1.174973e-09 | 2.216000e-07 | SP100, SMPD1, MX1, ISG15, IFIT1, SHFL |

**Table S10. Top 10 Significant P-Values and Q-Values for GO Biological Process 2023 in Downregulated Significant Genes When Comparing MPV-None-72 to Mock-72.**

| **Term** | **p-value** | **q-value** | **Overlap genes** |
| --- | --- | --- | --- |
| Regulation Of Neutrophil Migration (GO:1902622) | 0.000262 | 0.159959 | RAC2, OLFM4 |
| Regulation Of Cytoplasmic Transport (GO:1903649) | 0.000511 | 0.159959 | MAP2K2, SRC |
| Formation Of Cytoplasmic Translation Initiation Complex (GO:0001732) | 0.000840 | 0.159959 | EIF3M, EIF3H |
| G Protein-Coupled Acetylcholine Receptor Signaling Pathway (GO:0007213) | 0.001248 | 0.159959 | GNA15, GRK2 |
| Regulation Of Early Endosome To Late Endosome Transport (GO:2000641) | 0.001563 | 0.159959 | MAP2K2, SRC |
| Regulation Of Centrosome Cycle (GO:0046605) | 0.002294 | 0.159959 | NPM1, CCNL2 |
| Negative Regulation Of Protein Kinase Activity (GO:0006469) | 0.002581 | 0.159959 | DBNDD1, NPM1, CHP1 |
| Cytoplasmic Translation (GO:0002181) | 0.003019 | 0.159959 | RPL4, EIF3M, RPL17 |
| Intracellular Protein Transport (GO:0006886) | 0.003335 | 0.159959 | AKIRIN2, RAB1A, NPM1, CHP1, STX10 |
| Cytoplasmic Translational Initiation (GO:0002183) | 0.003889 | 0.159959 | EIF3M, EIF3H |

**Table S11. Top 10 Significant P-Values and Q-Values for GO Biological Process 2023 in Upregulated Significant Genes When Comparing PIV3-None-72 to Mock-72.**

| **Term** | **p-value** | **q-value** | **Overlap genes** |
| --- | --- | --- | --- |
| Defense Response To Symbiont (GO:0140546) | 1.205983e-48 | 4.276417e-45 | IFITM3, RTP4, IFITM1, IFITM2, IFIT5, CGAS, DDX60L, IFIT1, IFI44L, IFIT3, IFIT2, OASL, PYCARD, IFIH1, TBK1, TRIM5, CASP1, DHX15, AZI2, IKBKE, GBP5, GBP7, RSAD2, NT5C3A, PLSCR1, AIM2, IFI27, OAS1, OAS2, IRF1, OAS3, IFIT1B, IRF2, IRF7, TLR3, TLR2, TRIM56, IFI6, DDX60, SAMHD1, TANK, IFNL2, IFNL1, IFI16, PMAIP1, IFNL3, APOBEC3C, APOBEC3F, APOBEC3G, MLKL, IFNB1, STAT1, MX2, STAT2, MX1, EIF2AK2, ISG15, BST2, ISG20, MOV10, ZNFX1, STING1, F2RL1, TRIM31, SHFL, APOBEC3A, MYD88, APOBEC3B |
| Defense Response To Virus (GO:0051607) | 6.670557e-48 | 1.182690e-44 | IFITM3, RTP4, IFITM1, IFITM2, IFIT5, CGAS, DDX60L, IFIT1, IFI44L, IFIT3, IFIT2, OASL, PYCARD, IFIH1, TBK1, TRIM5, DHX58, CASP1, DHX15, TRIM25, AZI2, TRIM21, IKBKE, NCK1, GBP5, GBP7, RSAD2, NT5C3A, PLSCR1, AIM2, IFI27, OAS1, OAS2, IRF1, OAS3, IFIT1B, IRF2, IRF7, TLR3, TLR2, TRIM56, IFI6, DDX60, SAMHD1, TANK, USP18, IFNL2, IFNL1, IFI16, PMAIP1, IFNL3, APOBEC3C, APOBEC3F, APOBEC3G, MLKL, IFNB1, STAT1, MX2, STAT2, MX1, EIF2AK2, ISG15, PML, BST2, ISG20, CXCL10, MOV10, ZNFX1, STING1, F2RL1, TRIM31, SHFL, APOBEC3A, MYD88, APOBEC3B |
| Negative Regulation Of Viral Process (GO:0048525) | 5.709537e-30 | 6.748673e-27 | IFITM3, IFITM1, IFITM2, IFIT5, ZC3HAV1, IFIT1, OASL, IFIH1, ZFP36, IFI16, CCL5, TRIM21, IFNL3, N4BP1, APOBEC3C, APOBEC3F, RSAD2, APOBEC3G, IFNB1, STAT1, MX1, EIF2AK2, ISG15, BST2, ISG20, FAM111A, PLSCR1, ZNFX1, OAS1, OAS2, OAS3, TRIM14, TRIM31, SHFL, APOBEC3A |
| Negative Regulation Of Viral Genome Replication (GO:0045071) | 1.669261e-28 | 1.479800e-25 | IFITM3, IFITM1, IFITM2, IFIT5, ZC3HAV1, IFIT1, OASL, RESF1, IFIH1, IFI16, CCL5, IFNL3, N4BP1, APOBEC3C, APOBEC3F, RSAD2, APOBEC3G, IFNB1, MX1, EIF2AK2, ISG15, BST2, ISG20, FAM111A, PLSCR1, ZNFX1, OAS1, OAS2, OAS3, SHFL, APOBEC3A, APOBEC3B |
| Regulation Of Viral Genome Replication (GO:0045069) | 3.590358e-24 | 2.546282e-21 | IFITM3, IFITM1, IFITM2, STAU1, IFIT5, ZC3HAV1, IFIT1, OASL, IFIH1, IFI16, CCL5, IFNL3, N4BP1, APOBEC3C, GBP7, APOBEC3F, RSAD2, APOBEC3G, IFNB1, MX1, EIF2AK2, ISG15, BST2, ISG20, FAM111A, PLSCR1, ZNFX1, OAS1, OAS2, OAS3, SHFL, APOBEC3A |
| Response To Cytokine (GO:0034097) | 3.350446e-18 | 1.980114e-15 | IFITM3, CD274, IFITM1, SP100, IFITM2, CALCOCO2, ADAR, CXCL16, NUB1, IRAK2, LAMP3, UBD, CASP3, LGALS9, JAK2, TRIM21, IKBKE, MCL1, GCH1, IL1R1, STAT1, MX2, MX1, EIF2AK2, LIFR, ISG15, IRAK3, OSMR, PML, BST2, CH25H, PLSCR1, XAF1, SHFL, MYD88, TRIM56 |
| Regulation Of Innate Immune Response (GO:0045088) | 3.068497e-16 | 1.554413e-13 | CFH, CGAS, TNFAIP3, XIAP, IFI35, SAMHD1, IFI16, CCL5, DHX58, TRIM21, N4BP1, GBP5, AKIRIN2, IFNB1, TREX1, ERAP1, TRAFD1, IRAK3, PARP9, EREG, HLA-E, PLSCR1, IRF1, ADAM8, BIRC2, BIRC3, TRIM56 |
| Positive Regulation Of Intracellular Signal Transduction (GO:1902533) | 5.735796e-14 | 2.542392e-11 | RET, BCAR3, BMPR2, PLEKHF1, NCF1, RNF13, RTKN2, HTR2B, IFIT5, SECTM1, IFI35, FGF2, CX3CL1, BBC3, PYCARD, TBK1, CASP10, TRIM5, FAM110C, TNFSF10, DHX15, CASP1, TRIM25, LGALS9, SOX9, JAK2, IKBKE, TRIM21, TRIM22, ARRDC3, SHISA5, TICAM1, DDIT3, PELI1, ADAM8, RBCK1, NUPR1, BIRC2, TLR3, HBEGF, BIRC3, TRIM56, AVPI1, TLR2, NOD2, ADRB2, CCL5, UBD, DHX36, MIER1, PMAIP1, RICTOR, IGFBP6, APOL2, LYN, TCF7L2, NDFIP1, TGFB1, DAB2IP, LIF, EIF2AK2, SEPTIN4, CFLAR, BST2, AXL, F2RL1, TRIM38, PIK3AP1, MYD88, TRIM34 |
| Regulation Of I-kappaB kinase/NF-kappaB Signaling (GO:0043122) | 7.258738e-14 | 2.859943e-11 | IFIT5, HTR2B, SECTM1, TNFAIP3, RORA, NOD2, TANK, CX3CL1, PYCARD, TBK1, CASP10, TRIM5, UBD, DHX36, MIER1, TNFSF10, DHX15, CASP1, TRIM25, LGALS9, TRIM21, IKBKE, TRIM22, APOL2, GBP7, NDFIP1, TLE1, STAT1, DAB2IP, SHISA5, NR1D1, CFLAR, TICAM1, BST2, PELI1, F2RL1, RBCK1, TRIM38, OPTN, MYD88, BIRC2, BIRC3, TRIM34 |
| Response To Type II Interferon (GO:0034341) | 5.049963e-13 | 1.790717e-10 | IFITM3, GBP6, GBP5, IFITM1, SP100, IFITM2, GCH1, CALCOCO2, STAT1, CX3CL1, CXCL16, BST2, NUB1, CCL5, UBD, CASP1, LGALS9, CD47, TRIM21, GBP1, SHFL, GBP4, HLA-DPA1, TLR2 |

**Table S12. Top 10 Significant P-Values and Q-Values for GO Biological Process 2023 in Downregulated Significant Genes When Comparing PIV3-None-72 to Mock-72.**

| **Term** | **p-value** | **q-value** | **Overlap genes** |
| --- | --- | --- | --- |
| Translation (GO:0006412) | 3.641383e-65 | 1.185634e-61 | RPL4, RPL5, RPL30, RPL3, RPL32, RPL31, RPL34, RPL8, APEH, RPL10A, RPL9, RPL6, RPL7, EEF1B2, RPS14, RPS17, RPS16, RPL18A, RPS18, RPL38, RPL37, RPS11, RPS10, RARS2, RARS1, RPL21, RPS7, RPS8, RPS5, RPL22, RPS6, RPSA, TUFM, EEF1A1, LARS1, RPL27, RPL26, AURKAIP1, RPL29, UBA52, DAP3, MRPL12, RPS4Y1, EIF4EBP2, VARS1, MRPL23, EEF2, HARS2, RPS26, EPRS1, RPL27A, RPS20, RPS21, RPS24, FARSB, MRPS15, RPLP1, RPLP0, MRPL36, MRPL37, MRPL34, RPS4X, MRPL3, RPL7A, RACK1, RPLP2, MRPS28, MRPS24, PABPC4, MRPS2, RPL13A, RPS3A, MRPL45, MRPS6, EEF1G, SARS1, MRPL51, EEF1D, RPL37A, RPL10, RPL12, RPL11, MRPL55, RPS15A, RPS3, RPL13, IARS2, RPL15, RPS2, IARS1, RPL18, RPS27A, RPL17, RPL19, GADD45GIP1, RWDD1, GARS1, ABCE1 |
| Cytoplasmic Translation (GO:0002181) | 1.380691e-56 | 2.247765e-53 | RPL4, RPL5, RPL30, RPL3, RPL32, RPL31, RPL34, RPLP1, RPLP0, RPL10A, RPL8, RPL9, RPL6, RPL7, RPS4X, RPL7A, RPS14, RPS17, RPS16, RPL18A, RPS18, RACK1, RPLP2, RPL38, RPL37, RPS11, RPS10, RPL21, RPS7, RPS8, RPS5, RPL22, RPS6, RPL13A, RPS3A, RPSA, SARS1, RPL37A, RPL27, RPL26, RPL29, UBA52, RPL10, RPL12, RPL11, RPS15A, RPS3, RPL13, RPL15, RPS2, RPL18, RPS27A, RPL17, RPL19, RWDD1, RPS26, EIF3M, RPL27A, RPS20, RPS21, RPS24 |
| Macromolecule Biosynthetic Process (GO:0009059) | 3.186957e-56 | 3.458910e-53 | RPL4, RPL5, RPL30, MRPS15, RPL3, RPL32, RPL31, RPL34, RPLP1, RPLP0, MRPL36, MRPL37, RPL10A, RPL8, MRPL34, RPL9, RPL6, RPL7, EEF1B2, RPS4X, RPS14, MRPL3, RPL7A, RPS17, RPS16, RPL18A, RPS18, RPLP2, RPL38, RPL37, RPS11, RPS10, RPL21, RPS7, RPS8, RPS5, PABPC4, RPL22, RPS6, RPL13A, RPSA, RPS3A, MRPS6, TUFM, EEF1A1, EEF1G, SARS1, MRPL51, EEF1D, RPL37A, RPL27, RPL26, RPL29, RPL10, RPL12, RPL11, MRPL12, RPS4Y1, MRPL55, RPS15A, POLD2, RPS3, EIF4EBP2, RPL13, APOE, RPL15, RPS2, RPL18, RPS27A, RPL17, RPL19, EEF2, MRPL23, HARS2, RPS26, RPL27A, RPS20, RPS21, EIF4G2, RPS24, FARSB |
| Peptide Biosynthetic Process (GO:0043043) | 2.186129e-52 | 1.779509e-49 | RPL4, RPL5, RPL30, MRPS15, RPL3, RPL32, RPL31, RPL34, RPLP1, RPLP0, MRPL36, MRPL37, RPL10A, RPL8, MRPL34, RPL9, RPL6, RPL7, RPS4X, RPS14, MRPL3, RPL7A, RPS17, RPS16, RPL18A, RPS18, RPLP2, RPL38, RPL37, RPS11, RPS10, RPL21, RPS7, RPS8, RPS5, PABPC4, RPL22, RPS6, RPL13A, RPSA, RPS3A, MRPS6, EEF1A1, SARS1, MRPL51, RPL37A, RPL27, RPL26, RPL29, RPL10, RPL12, RPL11, MRPL12, RPS4Y1, MRPL55, RPS15A, RPS3, EIF4EBP2, RPL13, RPL15, RPS2, RPL18, RPS27A, RPL17, RPL19, MRPL23, HARS2, RPS26, RPL27A, RPS20, RPS21, RPS24, FARSB |
| Gene Expression (GO:0010467) | 4.488870e-35 | 2.923152e-32 | RPL4, RPL5, RPL30, MRPS15, RPL3, RPL32, RPL31, RPL34, RPLP1, RPLP0, MRPL36, MRPL37, RPL8, MRPL34, RPL10A, RPL9, RPL6, RPL7, RBM3, RPS4X, RPS14, MRPL3, RBM4, RPL7A, RPS17, RPS16, RPL18A, RPS18, RPLP2, RPL38, RPL37, RPS11, RPS10, RPL21, RPS7, RPS8, PABPC4, RPS5, RPL22, RPS6, RPL13A, RPSA, RPS3A, MRPS6, EEF1A1, SARS1, MRPL51, RPL37A, RPL27, RPL26, RPL29, RPL10, RPL12, RPL11, MRPL12, RPS4Y1, MRPL55, RPS15A, RPS3, RPL13, EIF4EBP2, RPL15, RPS2, RPL18, RPS27A, RPL17, RPL19, PRSS37, MRPL23, HARS2, SORL1, RPS26, RPL27A, RPS20, STUB1, RPS21, RPS24, FARSB |
| Regulation Of Translation (GO:0006417) | 2.564027e-11 | 1.391412e-08 | RPL5, DDX6, RPL10, PRKDC, HSPB1, MSI2, YBX1, BZW2, FXR1, RBM3, RPS4X, RBM4, ENC1, RACK1, RPS3, METTL5, EIF4B, NANOS1, EIF2B4, NPM1, PRMT1, ELP2, RPL13A, EEF2, LRPPRC, MTOR, EIF6, EPRS1, SERBP1, EIF3H, EIF3E, RPL26, VIM, GAPDH, EIF3D, EIF4G2 |
| Aerobic Respiration (GO:0009060) | 3.032854e-10 | 1.410711e-07 | NDUFA13, NDUFB7, NDUFA6, NDUFB10, UQCRB, NDUFB11, MDH2, NDUFA10, NDUFA2, NDUFA1, CHCHD10, UQCRH, OXA1L, NDUFS5, NDUFAB1, UQCRC1, MTFR1, NDUFV1 |
| protein-RNA Complex Assembly (GO:0022618) | 6.239275e-09 | 2.539385e-06 | RPL5, HSP90AB1, RPL10, PRKDC, RPLP0, RPL11, RPL6, RPS14, SNRPD2, PUF60, RPL38, TXNL4A, XRCC5, RPS5, RPSA, NUDT21, EIF3M, EIF2S3, EIF6, EIF3K, EIF3L, AGO1, STRAP, EIF3H, EIF3E, EIF3D, NOP53 |
| Cellular Respiration (GO:0045333) | 2.972301e-08 | 1.075312e-05 | NDUFA13, NDUFB7, NDUFA6, NDUFB10, UQCRB, NDUFB11, MDH2, COX4I1, NDUFA10, NDUFA2, NDUFA1, ETFB, UQCRH, OXA1L, NDUFS5, NDUFAB1, UQCRC1, MTFR1, NDUFV1 |
| Positive Regulation Of Protein Metabolic Process (GO:0051247) | 4.450282e-08 | 1.449012e-05 | RPL5, NDUFA13, HDAC2, PRKDC, FAF1, RAB1B, FXR1, RBM3, RPS4X, METTL5, APOE, MAP2K5, NPM1, PRMT1, WFS1, IL18, WNT7A, EEF2, SORL1, MTOR, DDB1, EIF6, EIF3E, STUB1, RPL26, VIM, AURKAIP1, SEC22B, EIF3D, VPS28 |

**Table S13. Top 10 Significant P-values and Q-values for GO Biological Process 2023 in Upregulated Significant Genes Common to IAV/MPV/PIV3 vs. Mock at 24 Hours Post-infection.**

| **Term** | **p-value** | **q-value** | **Overlap genes** |
| --- | --- | --- | --- |
| Defense Response To Symbiont (GO:0140546) | 3.055391e-22 | 5.102503e-20 | IFITM1, PLSCR1, RSAD2, OAS1, OAS2, MX2, OAS3, MX1, ISG15, IFIT1, IFIT3, IFIT2 |
| Defense Response To Virus (GO:0051607) | 6.289549e-21 | 5.251774e-19 | IFITM1, PLSCR1, RSAD2, OAS1, OAS2, MX2, OAS3, MX1, ISG15, IFIT1, IFIT3, IFIT2 |
| Negative Regulation Of Viral Genome Replication (GO:0045071) | 1.501452e-19 | 8.358081e-18 | IFITM1, PLSCR1, RSAD2, OAS1, OAS2, OAS3, MX1, ISG15, IFIT1 |
| Negative Regulation Of Viral Process (GO:0048525) | 5.856740e-19 | 2.445189e-17 | IFITM1, PLSCR1, RSAD2, OAS1, OAS2, OAS3, MX1, ISG15, IFIT1 |
| Regulation Of Viral Genome Replication (GO:0045069) | 1.440569e-18 | 4.811501e-17 | IFITM1, PLSCR1, RSAD2, OAS1, OAS2, OAS3, MX1, ISG15, IFIT1 |
| Antiviral Innate Immune Response (GO:0140374) | 4.308594e-10 | 1.199225e-08 | OAS1, MX1, IFIT1, IFIT3, IFIT2 |
| Response To Cytokine (GO:0034097) | 9.218174e-10 | 2.199193e-08 | IFITM1, PLSCR1, MX2, MX1, ISG15, XAF1 |
| Regulation Of Ribonuclease Activity (GO:0060700) | 6.112990e-09 | 1.134299e-07 | OAS1, OAS2, OAS3 |
| Interleukin-27-Mediated Signaling Pathway (GO:0070106) | 6.112990e-09 | 1.134299e-07 | OAS1, OAS2, MX1 |
| Response To Interferon-Beta (GO:0035456) | 1.075154e-08 | 1.795507e-07 | IFITM1, PLSCR1, OAS1, XAF1 |

**Table S14. Top 10 Significant P-values and Q-values for GO Biological Process 2023 in Upregulated Significant Genes Common to IAV/MPV/PIV3 vs. Mock at 72 Hours Post-infection.**

| **Term** | **p-value** | **q-value** | **Overlap genes** |
| --- | --- | --- | --- |
| Defense Response To Virus (GO:0051607) | 1.207965e-52 | 1.211589e-49 | IFITM3, RTP4, IFITM1, IFI6, IFIT5, DDX60L, IFIT1, DDX60, SAMHD1, USP18, IFI44L, IFIT3, IFIT2, OASL, IFIH1, IFI16, DHX58, TRIM25, TRIM21, RSAD2, STAT1, MX2, MX1, EIF2AK2, ISG15, PML, ISG20, NT5C3A, BST2, CXCL10, PLSCR1, ZNFX1, IFI27, OAS1, OAS2, IRF1, OAS3, IFIT1B, IRF7, SHFL, APOBEC3A, TLR3 |
| Defense Response To Symbiont (GO:0140546) | 1.588775e-46 | 7.967708e-44 | IFITM3, RTP4, IFITM1, IFI6, IFIT5, DDX60L, IFIT1, DDX60, SAMHD1, IFI44L, IFIT3, IFIT2, OASL, IFIH1, IFI16, RSAD2, STAT1, MX2, MX1, EIF2AK2, ISG15, ISG20, NT5C3A, BST2, PLSCR1, ZNFX1, IFI27, OAS1, OAS2, IRF1, OAS3, IFIT1B, IRF7, SHFL, APOBEC3A, TLR3 |
| Negative Regulation Of Viral Process (GO:0048525) | 7.229291e-35 | 2.416993e-32 | IFITM3, IFITM1, RSAD2, STAT1, MX1, IFIT5, EIF2AK2, ISG15, ZC3HAV1, IFIT1, OASL, IFIH1, BST2, ISG20, PLSCR1, ZNFX1, OAS1, IFI16, OAS2, OAS3, TRIM21, SHFL, APOBEC3A |
| Negative Regulation Of Viral Genome Replication (GO:0045071) | 1.889924e-32 | 4.738984e-30 | IFITM3, IFITM1, RSAD2, MX1, IFIT5, EIF2AK2, ISG15, ZC3HAV1, IFIT1, OASL, IFIH1, BST2, ISG20, PLSCR1, ZNFX1, OAS1, IFI16, OAS2, OAS3, SHFL, APOBEC3A |
| Regulation Of Viral Genome Replication (GO:0045069) | 7.135177e-30 | 1.431317e-27 | IFITM3, IFITM1, RSAD2, MX1, IFIT5, EIF2AK2, ISG15, ZC3HAV1, IFIT1, OASL, IFIH1, BST2, ISG20, PLSCR1, ZNFX1, OAS1, IFI16, OAS2, OAS3, SHFL, APOBEC3A |
| Response To Cytokine (GO:0034097) | 4.515305e-19 | 7.548085e-17 | IFITM3, IFITM1, SP100, IL1R1, STAT1, MX2, MX1, EIF2AK2, ISG15, ADAR, PML, BST2, PLSCR1, LAMP3, LGALS9, XAF1, TRIM21, SHFL |
| Response To Interferon-Beta (GO:0035456) | 2.920577e-15 | 4.184770e-13 | IFITM3, BST2, IFITM1, PLSCR1, IFI16, OAS1, STAT1, IRF1, XAF1, SHFL |
| Antiviral Innate Immune Response (GO:0140374) | 1.126368e-14 | 1.412183e-12 | IFIH1, CXCL10, OAS1, DHX58, MX1, TRIM25, EIF2AK2, IFIT1, USP18, IFIT3, IFIT2 |
| Response To Type II Interferon (GO:0034341) | 7.265284e-12 | 8.096756e-10 | IFITM3, BST2, IFITM1, SP100, STAT1, LGALS9, CD47, TRIM21, GBP1, SHFL, GBP4 |
| Interleukin-27-Mediated Signaling Pathway (GO:0070106) | 1.401159e-11 | 1.405363e-09 | OAS1, OAS2, STAT1, MX1, OASL |


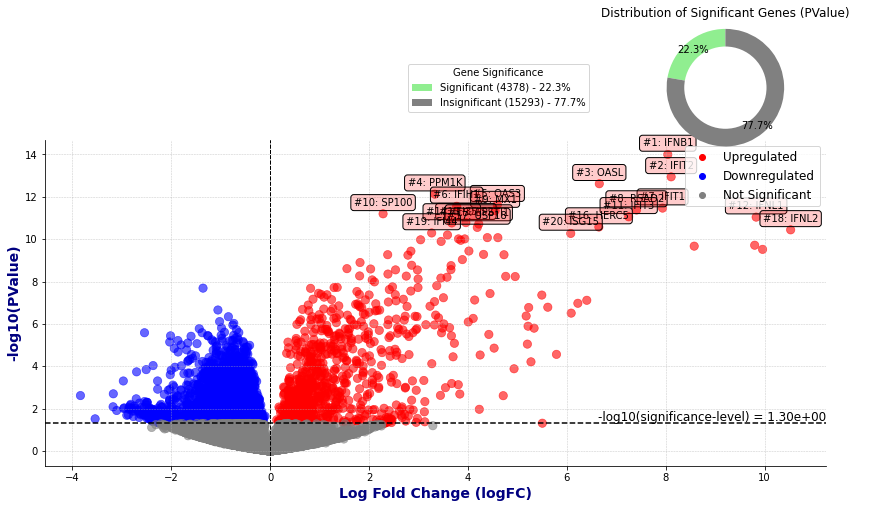


***Figure S1(a):*** *Volcano Plots and Analysis for IAV-Infected OTEs compared to Mock infection at 24 hours post-infection.*
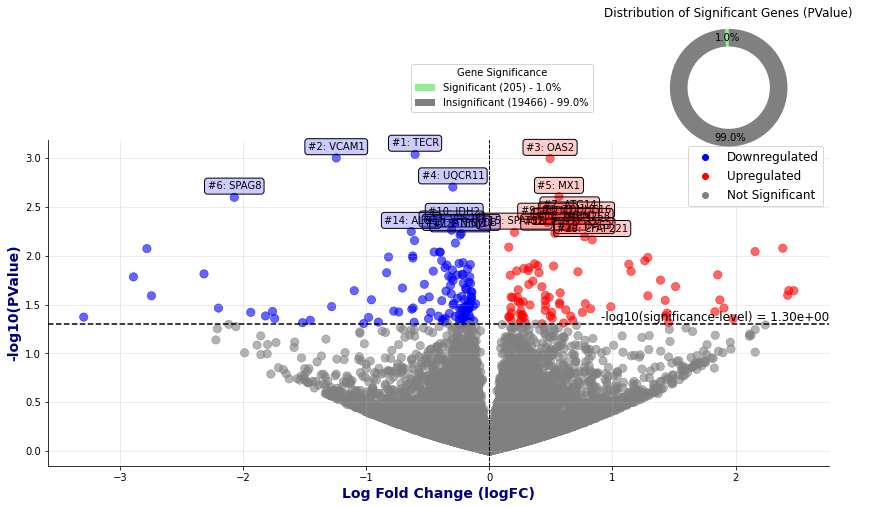


***Figure S1(b):*** *Volcano Plots and Analysis for MPV-Infected OTEs compared to Mock infection at 24 hours post-infection.*


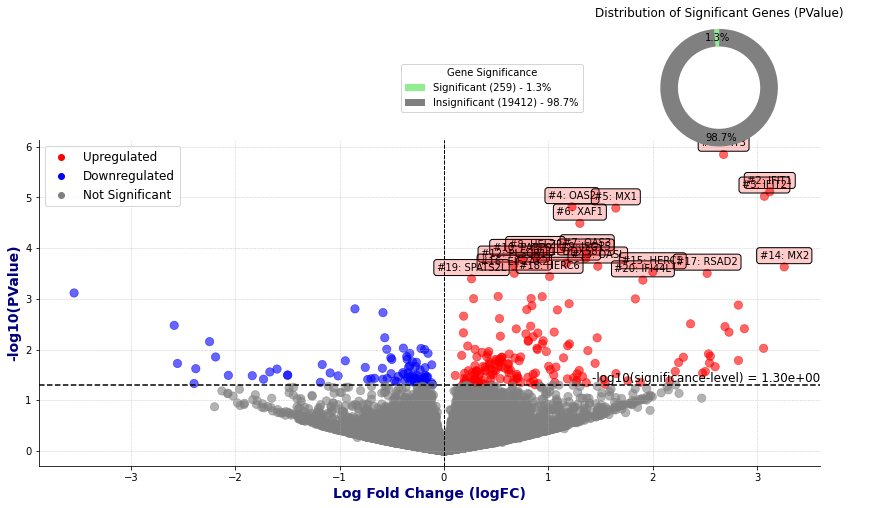


***Figure S1(c):*** *Volcano Plots and Analysis for PIV3-Infected OTEs compared to Mock infection at 24 hours post-infection.*


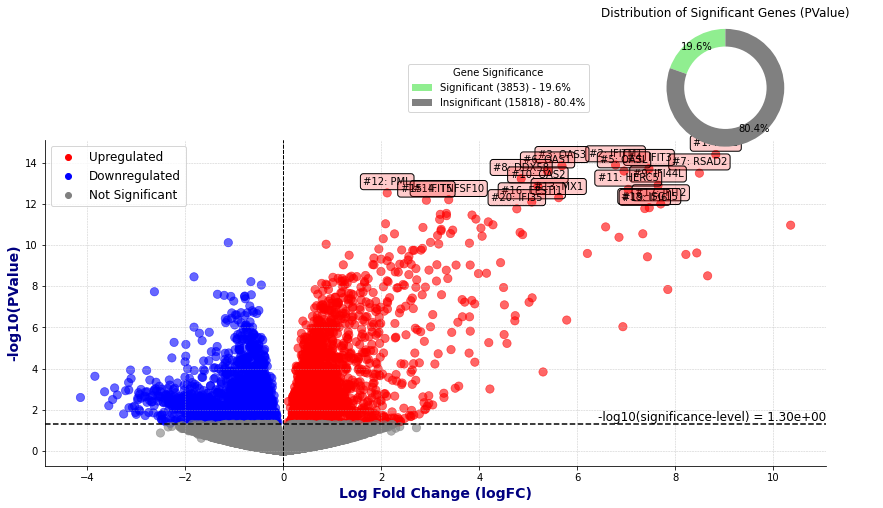


***Figure S1(d):*** *Volcano Plots and Analysis for IAV-Infected OTEs compared to Mock infection at 72 hours post-infection.*


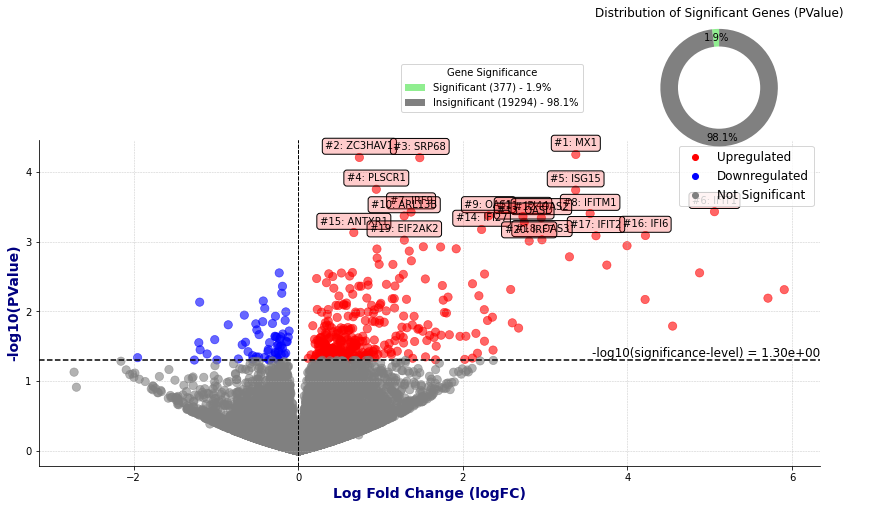


***Figure S1(e):*** *Volcano Plots and Analysis for MPV-Infected OTEs compared to Mock infection at 72 hours post-infection.*


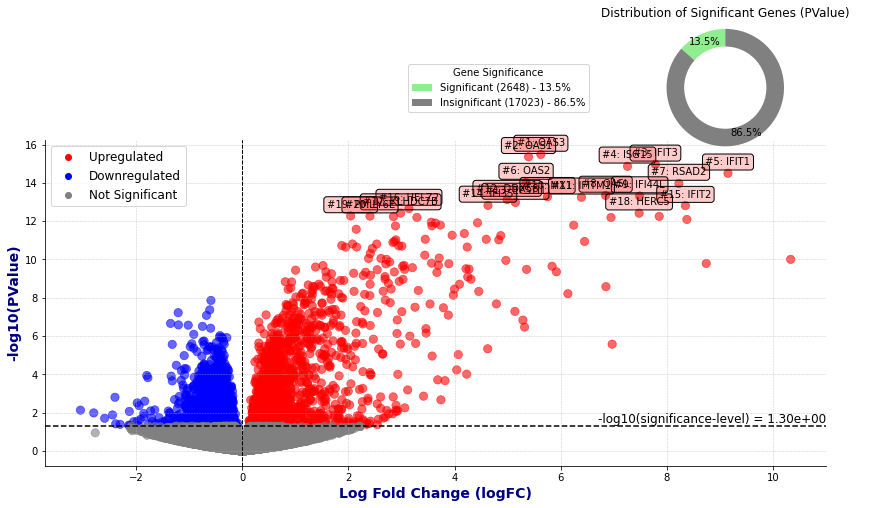


***Figure S1(f):*** *Volcano Plots and Analysis for PIV3-Infected OTEs compared to Mock infection at 72 hours post-infection.*


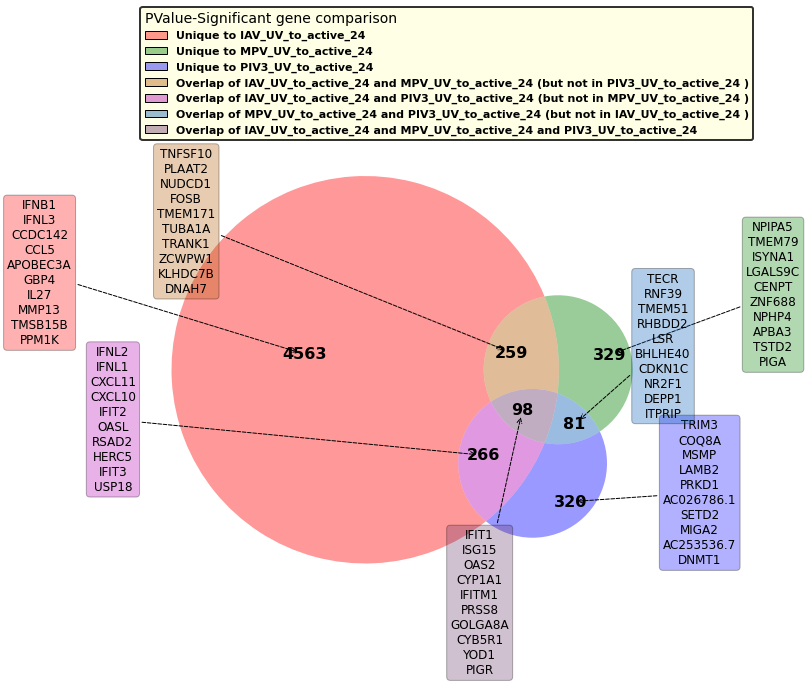


***Figure S2(a).*** *Venn Diagram of Differentially Expressed Genes at 24 Hours Post-Infection. This diagram illustrates the overlap and unique DEGs among samples infected with IAV, MPV, and PIV3 compared to UV-treated controls at 24 hours post-infection. Each circle represents a set of DEGs associated with a specific viral infection, with overlapping areas indicating common DEGs across different viruses. The numbers indicate the count of DEGs unique to each condition or shared between conditions, highlighting the gene expression patterns that are specific to each viral treatment and those that are commonly induced by different viruses at the early stage of infection.*


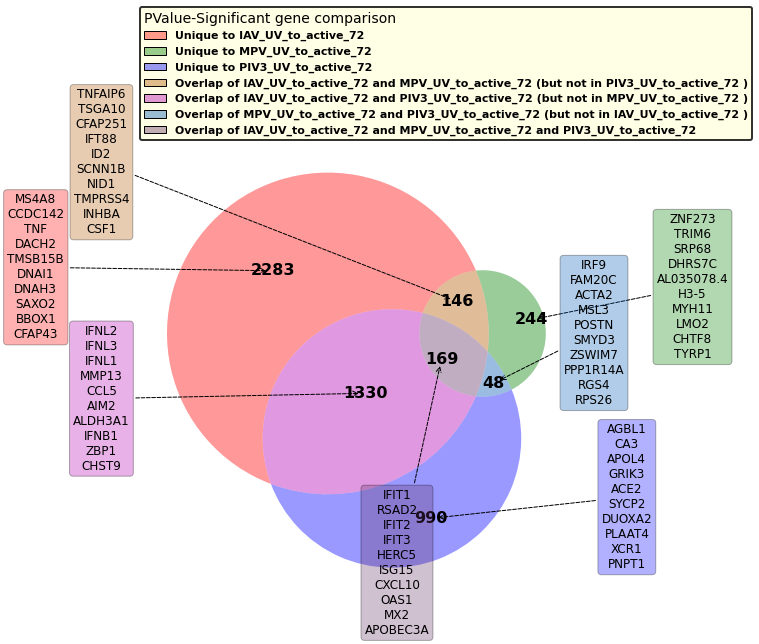


***Figure S2(b).*** *Venn Diagram of Differentially Expressed Genes at 72 Hours Post-Infection. This Venn diagram depicts the intersection and unique DEGs identified in OTE models infected with IAV, MPV, and PIV3 relative to UV-treated controls at 72 hours post-infection. Similar to the 24-hour time point, the circles correspond to DEGs for each virus, with the intersections revealing DEGs that are shared. The numbers reflect the DEGs that are exclusive to a given viral treatment or common between treatments, providing insights into the transcriptional responses that persist or emerge at the later stage of infection.*


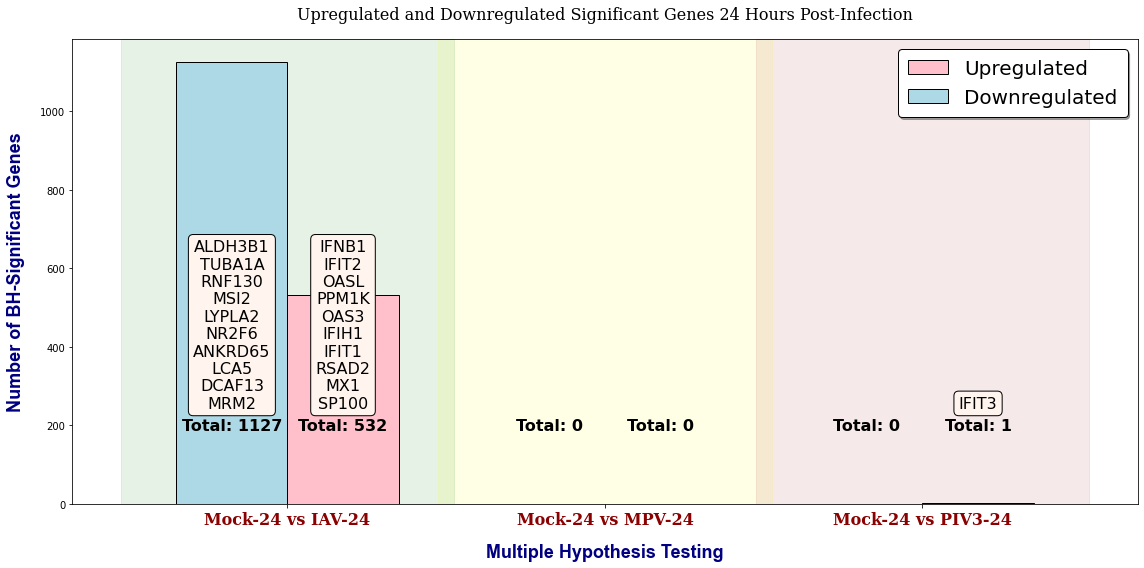
 **Figure S3(a).** Distribution of upregulated and downregulated significant genes in IAV, MPV, and PIV3 infected samples compared to Mock samples at **24 hours post-infection**, adjusted using the **Benjamini-Hochberg (BH)** method for controlling the false discovery rate.


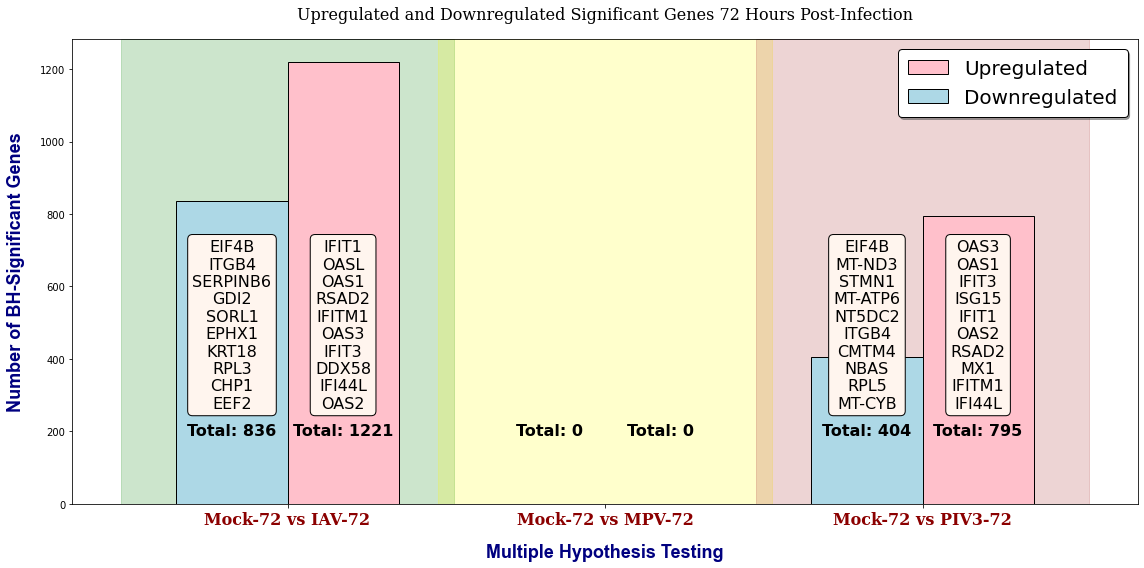
 **Figure S3(b).** Distribution of upregulated and downregulated significant genes in IAV, MPV, and PIV3 infected samples compared to Mock samples at **72 hours post-infection**, adjusted using the **Benjamini-Hochberg (BH)** method for controlling the false discovery rate.


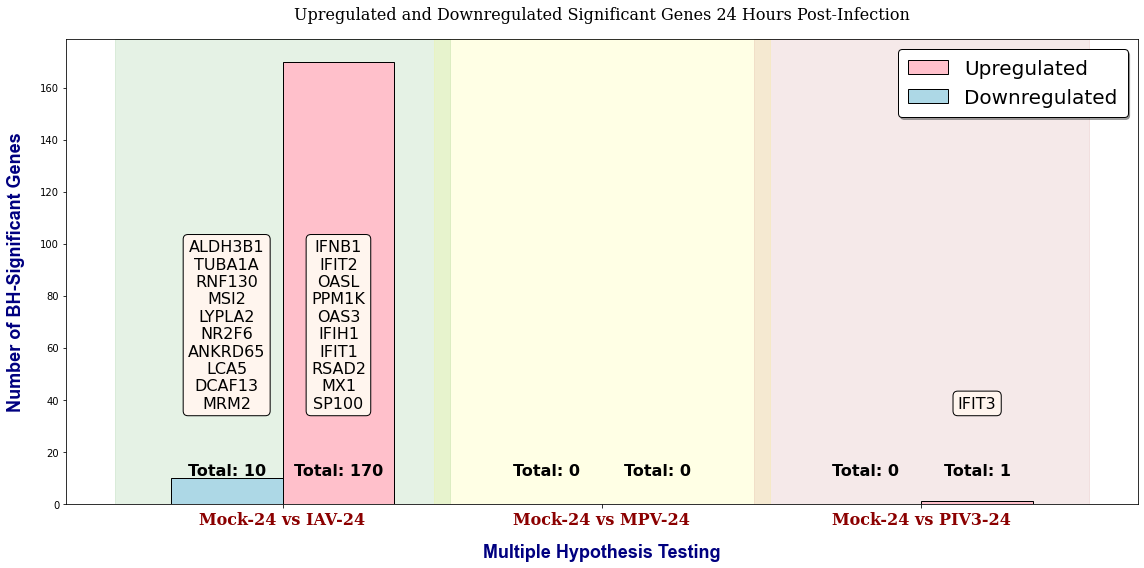
**Figure S4(a).** Distribution of upregulated and downregulated significant genes in IAV, MPV, and PIV3 infected samples compared to Mock samples at **24 hours post-infection**, using the **Bonferroni correction** to adjust for multiple comparisons.


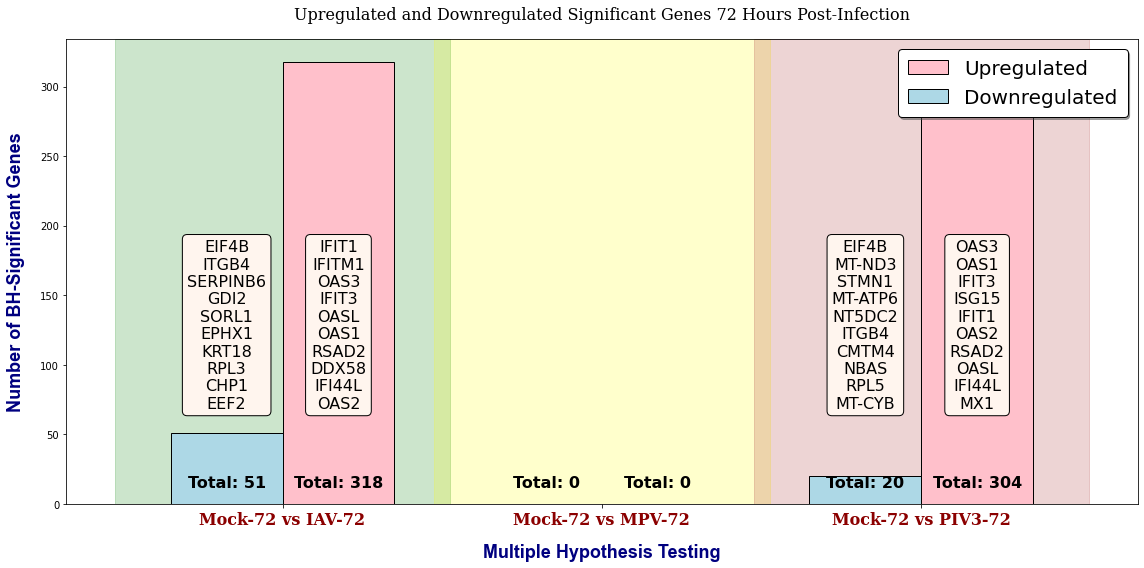


**Figure S4(b).** Distribution of upregulated and downregulated significant genes in IAV, MPV, and PIV3 infected samples compared to Mock samples at **72 hours post-infection**, using the **Bonferroni correction** to adjust for multiple comparisons.

| 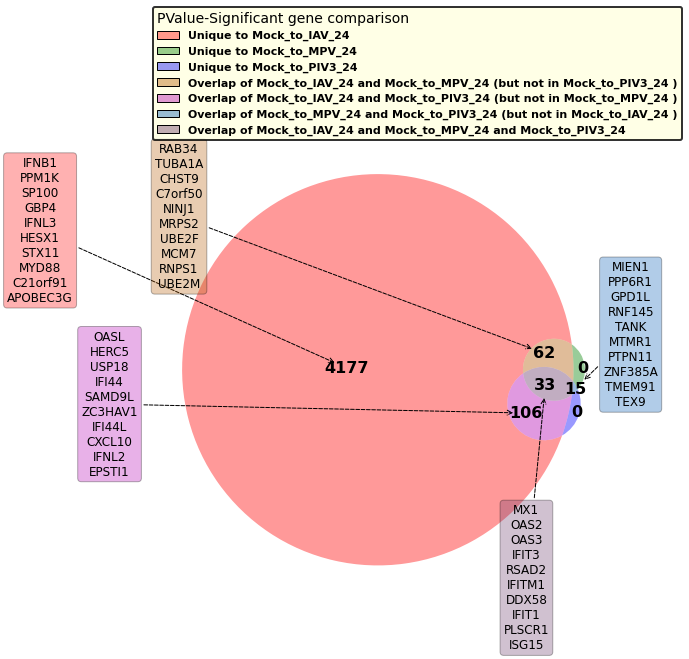  **Figure S5(a).** Venn diagram illustrating the overlap of BH significant genes in response to IAV, MPV, and PIV3 infection compared to Mock samples at 24 hours post-infection. | 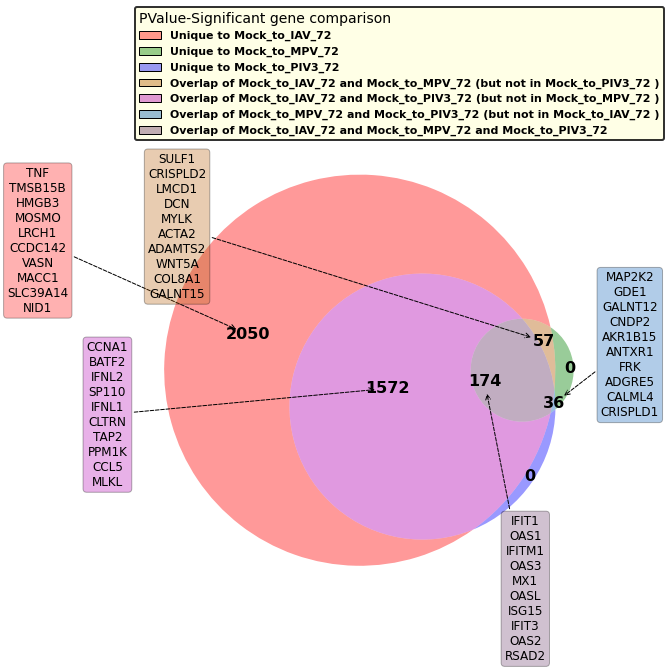  **Figure S5(b).** Venn diagram illustrating the overlap of BH significant genes in response to IAV, MPV, and PIV3 infection compared to Mock samples at 72 hours post-infection. |
| --- | --- |
| 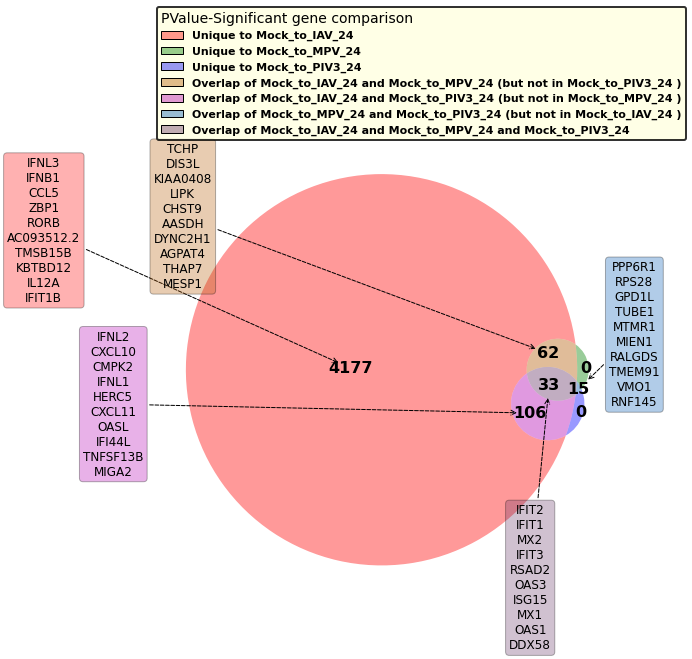  **Figure S5(c).** Venn diagram illustrating the overlap of Bonferroni significant genes in response to IAV, MPV, and PIV3 infection compared to Mock samples at 24 hours post-infection. | 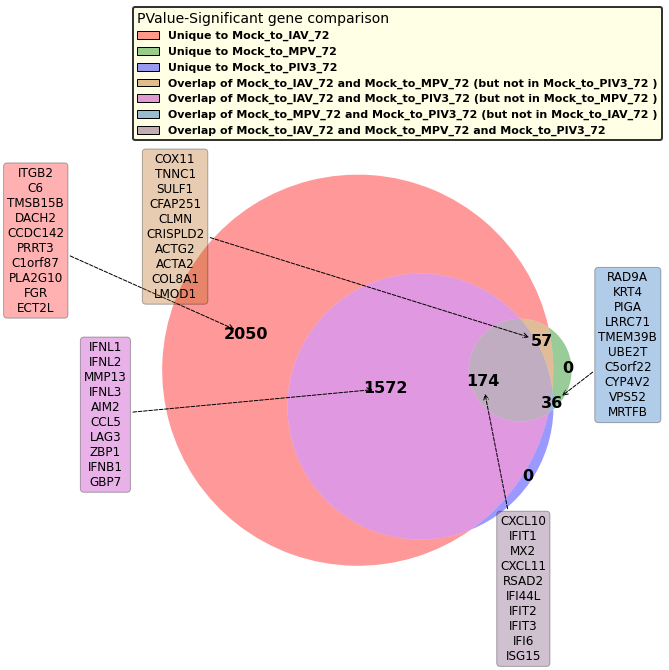  **Figure S5(d).** Venn diagram illustrating the overlap of Bonferroni significant genes in response to IAV, MPV, and PIV3 infection compared to Mock samples at 72 hours post-infection. |
